# Crypt fusion as a homeostatic mechanism in the human colon

**DOI:** 10.1136/gutjnl-2018-317540

**Published:** 2019-03-14

**Authors:** Ann-Marie Baker, Calum Gabbutt, Marc J Williams, Biancastella Cereser, Noor Jawad, Manuel Rodriguez-Justo, Marnix Jansen, Chris P Barnes, Benjamin D Simons, Stuart AC McDonald, Trevor A Graham, Nicholas A Wright

**Affiliations:** 1 Centre for Tumour Biology, Barts Cancer Institute, Queen Mary University of London, London, UK; 2 Department of Cell and Developmental Biology, University College London, London, UK; 3 Histopathology, University College London, London, UK; 4 Cavendish Laboratory, Department of Physics, University of Cambridge, Cambridge, UK; 5 The Wellcome Trust/Cancer Research UK Gurdon Institute, University of Cambridge, Cambridge, UK

**Keywords:** colon crypt, evolutionary dynamics, lineage tracing, crypt fission, crypt fusion, mathematical modelling

## Abstract

**Objective:**

The crypt population in the human intestine is dynamic: crypts can divide to produce two new daughter crypts through a process termed crypt fission, but whether this is balanced by a second process to remove crypts, as recently shown in mouse models, is uncertain. We examined whether crypt fusion (the process of two neighbouring crypts fusing into a single daughter crypt) occurs in the human colon.

**Design:**

We used somatic alterations in the gene cytochrome c oxidase (CCO) as lineage tracing markers to assess the clonality of bifurcating colon crypts (n=309 bifurcating crypts from 13 patients). Mathematical modelling was used to determine whether the existence of crypt fusion can explain the experimental data, and how the process of fusion influences the rate of crypt fission.

**Results:**

In 55% (21/38) of bifurcating crypts in which clonality could be assessed, we observed perfect segregation of clonal lineages to the respective crypt arms. Mathematical modelling showed that this frequency of perfect segregation could not be explained by fission alone (p<10^−20^). With the rates of fission and fusion taken to be approximately equal, we then used the distribution of CCO-deficient patch size to estimate the rate of crypt fission, finding a value of around 0.011 divisions/crypt/year.

**Conclusions:**

We have provided the evidence that human colonic crypts undergo fusion, a potential homeostatic process to regulate total crypt number. The existence of crypt fusion in the human colon adds a new facet to our understanding of the highly dynamic and plastic phenotype of the colonic epithelium.

**Significance of this study**

**What is already known on this subject?**

- Expansion of somatic mutations within the human colonic epithelium occurs through crypt fission, the process by which a parental crypt divides and produces two daughter crypts. Fission occurs at a low rate in the healthy adult colon; however, it is more frequent in certain disease states.
- As crypt density and colon length do not appear to increase over time, total crypt number must be regulated by a homeostatic process in which the fission-driven increase in crypt number is balanced by a process that decreases crypt number.
- A recent study has shown that, in the mouse intestine, two parental crypts can fuse into one daughter crypt in a process termed crypt fusion.

**What are the new findings?**

- Clonal lineage tracing analysis provides evidence for crypt fusion in the human colon, and suggests the rate of crypt fusion is balanced with that of crypt fission.
- Mathematical modelling that accounts for crypt fusion indicates that crypt fission occurs 20% more frequently (rate 0.011 divisions/crypt/year) than a previous estimation.

**How might it impact on clinical practice in the foreseeable future?**

- Crypt fusion may be a homeostatic mechanism to maintain intestinal epithelium integrity. Understanding the drivers of fusion could lead to new epithelial regeneration inducing therapies.
- Crypt fission is responsible for the spread of mutations in the early stages of colorectal tumourigenesis, and also for the regeneration of the colonic epithelium after injury. Consequently, manipulating the balance of crypt fission and fusion may represent a novel strategy for the prevention of the early expansion of precancerous mutant clones within the colonic epithelium.

## Introduction

The epithelial lining of the intestine is a highly dynamic population of rapidly renewing cells. The cells are organised into millions of small invaginations called crypts, and the base of each crypt houses a small population (<10) of actively dividing stem cells,^1^ the progeny of which migrate proximally along the crypt axis, become differentiated and are subsequently shed into the bowel lumen.^2 3^ In mice, the majority of cells within the small intestinal crypt are renewed every few days,^4^ while renewal of the human colon epithelium is measured to occur in slightly less than a week.^5^ Competition between cells for space in the stem cell niche located at the crypt base maintains homeostasis of cell number.^6–8^

The population of crypts is also dynamic. Crypt fission, the bifurcation of a parental crypt into two daughters, is responsible for postnatal expansion in crypt number^9^ and is observed to occur at a low rate in the healthy adult colon in both human and mouse.^3 10–12^ However, despite this low level fission—a growth process—there does not appear to be an increase in the total number of crypts in the intestine during ageing (the density of crypts and length of intestine do not appear to increase with age in mice^13^ or humans^14^) suggesting that the rate of crypt fission must be matched by an equal rate of crypt death. If, for example, every crypt in the colon divided only once in an adult lifetime, then the crypt number would double. Given that there is no direct histological evidence of crypt death in healthy colon (although it is clear that crypts can die and regrow in pathological conditions, such as in IBD^15^), it suggests that either crypt death is an extremely rapid process, or that some other hitherto unidentified homeostatic mechanism maintains crypt number. Recent measurements in mice have provided evidence for the latter: a combination of multicolour genetic labelling and intravital imaging showed the merging of two intestinal crypts (labelled with different colours) into a single new crypt over the course of a few days, a process that the authors termed *crypt fusion*.^13^ Furthermore, they showed that at a given timepoint postlabelling, 3.5% of labelled crypts were in fission and 4.1% were in fusion, suggesting that the two processes occur at approximately similar frequency.

Whether or not crypt fusion occurs in the human colon remains undetermined, as the transgenic labelling and live-imaging approach applied successfully in mice cannot be translated to humans. Instead, an approach that can be readily applied to human tissue is to exploit naturally occurring somatic mutations as lineage tracing markers, and examine the spatial distribution of mutant clones in tissue from older patients. Using this approach, we and others have shown that the distribution of crypt *patch size—*the number of adjacent crypts all bearing the same somatic mutation—increases with age in the colon^3 10 11^ providing evidence of ongoing crypt fission during ageing. Attempts to infer crypt death (or fusion) rates directly from these data requires longitudinal measurement; thus, an alternative approach is needed.

Here, we provide evidence of crypt fusion in the human colon. We have used naturally occurring somatic mutations that cause a histochemically detectable defect as clonal lineage tracing markers, and specifically analyse the clonal composition of branched crypts, which could be the intermediate products of either fission or fusion. We hypothesised that only crypts in fusion, and not those in fission, would be likely to show ‘perfect segregation’ of labelled and unlabelled lineages into the respective arms of the bifurcating crypt. We used mathematical modelling to assess the validity of this hypothesis given the data and show that only by allowing for fusion can we explain our observations. Finally, we revise the estimation of crypt fission rate in the human colon in the light of this new evidence for crypt fusion.

## Results

### Evidence for crypt fusion in human colon

We examined the spatial segregation of distinctly labelled clones within bifurcating crypts in human colon. Fresh frozen colonic mucosa was obtained from 13 patients (aged 39–79 years, patients 1–13 in table 1), including 7 people with familial adenomatous polyposis (FAP) or attenuated FAP (AFAP) who carry a germline pathogenic *APC* mutation, and 3 people with IBD. Both disease conditions are known to exhibit an increased rate of crypt bifurcation compared with healthy colon.^15 16^ Tissue was orientated such that the crypt axes were perpendicular to the section plane (*en face*) and sectioned serially from the luminal end of the crypt to the crypt base. Manual inspection of the serial sections led to the identification of a total of 309 ‘bifurcation events’ (59 721 crypts inspected, 0.52% bifurcating; table 1), where two crypts shared a region of epithelium. We observed an increased rate of bifurcation in diseased colon (normal colon=0.09%, AFAP/FAP colon=0.62%, IBD colon=1.44%, p=0.009 by the Kruskal-Wallis test) in agreement with previous reports,^15 16^ although with somewhat lower estimations of the proportion of bifurcating crypts. Previously, such bifurcation events were classified as a single crypt in the process of fission. However, based on static histological measures alone, these events could equally be associated with the fusion of neighbouring crypts. To discriminate between these possibilities, we considered whether temporal information could be inferred from clonal data.

**Table 1:** Patient details and raw bifurcation counts

Patient ref	Disease	Age	Total crypts	CCO+ crypts	CCO− crypts	CCO partial crypts	Total bifurcation events	Type I bifurcation events (% of total)	Type II bifurcation events (% of total)	Type III bifurcation events (% of total)	Fission/fusion rate (per crypt per year) (95% CI)	Duration of fission/fusion (weeks) (95% CI)
1	None	79	4919	4086	497	336	7	5 (71.4%)	2 (28.6%)	0	0.009 (0.007 to 0.011)	3.9 (3.1 to 4.7)
2	None	60	3547	3424	70	53	3	3 (100%)	0	0	0.005 (0.001 to 0.009)	4.1 (1.3 to 6.8)
3	None	64	6958	6798	101	59	5	5 (100%)	0	0	0.002 (0.001 to 0.003)	9.1 (2.5 to 15.7)
4	FAP	67	6417	5910	287	220	44	41 (93.2%)	1 (2.3%)	2 (4.5%)	0.017 (0.013 to 0.021)	10.7 (8.1 to 13.2)
5	FAP	59	4095	3713	193	189	23	20 (87.0%)	3 (13.0%)	0	0.033 (0.024 to 0.042)	4.4 (3.2 to 5.7)
6	FAP	39	3085	3018	43	24	48	48 (100%)	0	0	0.010 (0.002 to 0.019)	40.7 (6.8 to 74.6)
7a	AFAP	64	5448	5213	148	87	54	50 (92.6%)	3 (5.6%)	1 (1.9%)	0.012 (0.008 to 0.017)	20.9 (13.3 to 28.6)
7b	AFAP	64	5065	4463	291	311	25	20 (80.0%)	3 (12.0%)	2 (8.0%)	0.012 (0.008 to 0.015)	11.0 (8.0 to 14.0)
8	AFAP	65	11 755	11 351	213	191	25	18 (72.0%)	0	7 (28.0%)	0.014 (0.010 to 0.018)	3.9 (2.8 to 5.1)
9	AFAP	61	4174	3641	282	251	27	20 (74.1%)	2 (7.4%)	5 (18.5%)	0.027 (0.021 to 0.034)	6.2 (4.7 to 7.7)
10	AFAP	60	2038	1962	52	24	16	14 (87.5%)	1 (6.3%)	1 (6.3%)	0.011 (0.004 to 0.019)	17.1 (6.3 to 28.0)
11a	IBD	66	272	256	15	1	3	3 (100%)	0	0	ND	ND
11b	IBD	66	402	378	21	3	1	1 (100%)	0	0	ND	ND
12	IBD	72	301	238	58	5	3	1 (33.3%)	0	2 (66.7%)	ND	ND
13	IBD	65	1245	1057	102	86	25	22 (88.0%)	2 (8.0%)	1 (4.0%)	ND	ND
14	None	42	2702	ND	ND	ND	ND	ND	ND	ND	0.020 (0.006 to 0.034)	ND
15	None	50	2878	ND	ND	ND	ND	ND	ND	ND	0.009 (0.001 to 0.017)	ND
16	None	50	2883	ND	ND	ND	ND	ND	ND	ND	0.015 (0.006 to 0.024)	ND
17	None	62	12 030	ND	ND	ND	ND	ND	ND	ND	0.007 (0.004 to 0.010)	ND
18	None	65	29 839	ND	ND	ND	ND	ND	ND	ND	0.003 (0.002 to 0.004)	ND
19	None	72	4101	ND	ND	ND	ND	ND	ND	ND	0.005 (0.002 to 0.008)	ND
20	None	74	8792	ND	ND	ND	ND	ND	ND	ND	0.014 (0.011 to 0.017)	ND
21	None	74	22 045	ND	ND	ND	ND	ND	ND	ND	0.012 (0.010 to 0.014)	ND
22	None	80	6249	ND	ND	ND	ND	ND	ND	ND	0.014 (0.010 to 0.018)	ND
23	None	82	10 341	ND	ND	ND	ND	ND	ND	ND	0.016 (0.014 to 0.018)	ND
24	None	84	11 821	ND	ND	ND	ND	ND	ND	ND	0.024 (0.020 to 0.028)	ND

Patients 1–13 had serial sections available and were used to score crypt bifurcation frequencies.

Patients 14–24 had only a single section available and were used to assess CCO− patch size.

AFAP, attenuated familial adenomatous polyposis; CCO, cytochrome c oxidase; FAP, familial adenomatous polyposis; ND, not detemined.

To identify clonal lineages, we stained each specimen for cytochrome c oxidase (CCO) activity. Spontaneous loss of CCO activity (CCO−) is observed in the ageing human colon,^17^ and by age 80 approximately 30% of crypts show CCO deficiency^11^ that is readily visualised by enzyme histochemistry.^3 11 17^ CCO is a mitochondrially encoded gene, and CCO− is typically attributable to an underlying somatic mitochondrial DNA mutation.^17^ Single cell sequencing of the mitochondrial genome demonstrates the clonality of an expanded CCO− patch.^17^ Thus, loss of CCO activity provides a naturally occurring, easily visualised, clonal mark in human tissues.

**Figure 1:**
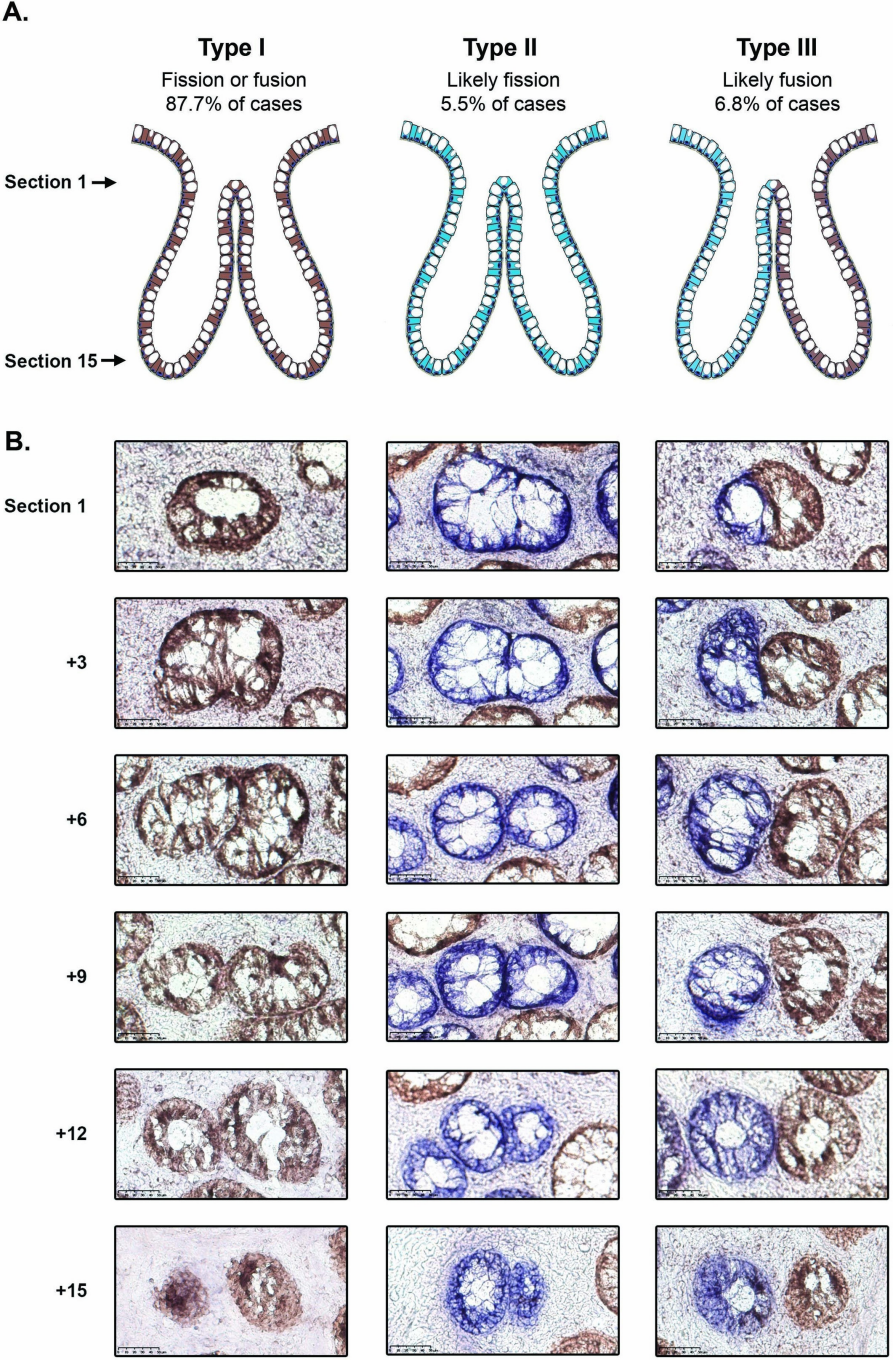
Analysis of cytochrome c oxidase (CCO) activity in bifurcating crypts. (A) Schematic diagram showing the distribution of CCO activity in type I, II and III bifurcation events. (B) Representative images of type I, II and III bifurcating crypts, with the upper row corresponding to the most luminal section and the lower row corresponding to the crypt base. The type I and III examples are taken from patient 8 and the type II example is from patient 4. Scale bars represent 50 μm.

The majority of bifurcating crypts (251/309; 81.2%) were entirely CCO-proficient (CCO+, ‘type I’, figure 1 and table 1); these were considered uninformative as they could be the intermediate product of either fission or fusion. Rare bifurcation events that involved partial CCO-deficient crypts (20/309, 6.5%) were also classed as ‘type I’ events. There were 17/309 (5.5%) of bifurcations that were entirely CCO-deficient (CCO−, ‘type II’, figure 1 and table 1), and we hypothesised that, due to the low frequency of CCO deficiency, these are highly likely to be the product of fission. The remaining 21/309 (6.8%) of bifurcations contained a mixture of CCO− and CCO+ cells, with the CCO+ and CCO− lineages, respectively restricted to single bifurcating crypt arms (‘type III’, figure 1, table 1 and online supplementary figure 1). We hypothesised that these ‘perfectly segregated bifurcating crypts’ are intermediates of the fusion of neighbouring CCO− (labelled) and CCO+ (unlabelled) crypts. Thus, out of a total of 38 bifurcating crypts where clonality could be accurately assessed, 21/38 (55%) were suggestive of fusion, and 17/38 (45%) were suggestive of fission.

10.1136/gutjnl-2018-317540.supp1 Supplementary data

We generated a three-dimensional (3D) reconstruction of a ‘type III’ bifurcation (online supplementary figure 2, supplementary videos 1 and 2) using digitised serial images of thin tissue sections. This detailed histological analysis shows that the bifurcating crypts analysed stayed closely associated through approximately half of the length of the crypt, forming one lumen close to the proximal surface. In the lower half of the crypts, the two bifurcated arms were clearly separated by stroma. The cellular contributions from each arm (respectively blue CCO− and brown CCO+ cells) show no intermixing in the shared proximal region. The lack of intermixing CCO− and CCO+ lineages at the proximal end of the merged crypt was also evident in a second crypt that we studied in detail (online supplementary video 3).

10.1136/gutjnl-2018-317540.supp4 Supplementary data

We took a frequentist hypothesis testing approach to evaluate the null hypothesis that these data could be explained by fission alone. We note that crypt death is not expected to cause crypt bifurcation, and so is irrelevant to the following mathematical argument. Specifically, we calculated the likelihood that a mixed CCO−/CCO+ parent crypt undergoing fission would, by chance, perfectly segregate the CCO− and CCO+ lineages into separate arms as it bifurcated. Our calculation assumed that a crypt contained a small number of stem cells (S) that were in neutral competition^3 6–8 10^ and that the rate at which new CCO− lineages developed was very small. On the initiation of fission, we assumed that stem cells were segregated in equal numbers to the two daughter crypts (S/2 stem cells into each daughter crypt), and so for perfect segregation of CCO− and CCO+ lineages to occur, exactly S/2 stem cells had to be CCO− at the moment that fission was initiated. Thus, we estimated the probability that a crypt would contain S/2 labelled stem cells on the initiation of fission, and used this value to calculate the chance of observing the 21 type III crypts among 309 bifurcating crypts (see online supplementary mathematical note). For the plausible range of S values (S=[5−10]), the probability of observing 21/309 perfectly segregated bifurcating crypts by fission alone was negligible (p<10^−20^ for all values of S; see online supplementary table 1). Hence, we rejected the null hypothesis and assumed a role for crypt fusion in explaining our data.

### Balanced rates of crypt fission and fusion

To determine if the rates of crypt fission and fusion are balanced in the human colon, we compared the number of ‘type II’ bifurcation events (likely fission) to the number of ‘type III’ bifurcation events (likely fusion). Assuming that the probability of observing a type II or type III event is proportional to the rate of fission or fusion, respectively, and that the amount of time a crypt undergoing fission or fusion spends in a bifurcating state is approximately equal, then if the fission and fusion rate were equal we would expect the number of type II or type III events to be similar. We find that the frequency of these events is similar (45% vs 55% of labelled bifurcations, respectively; table 1), supporting the idea of balanced rates of fission and fusion in the human colon.

### Revision of the crypt fission rate

**Figure 2:**
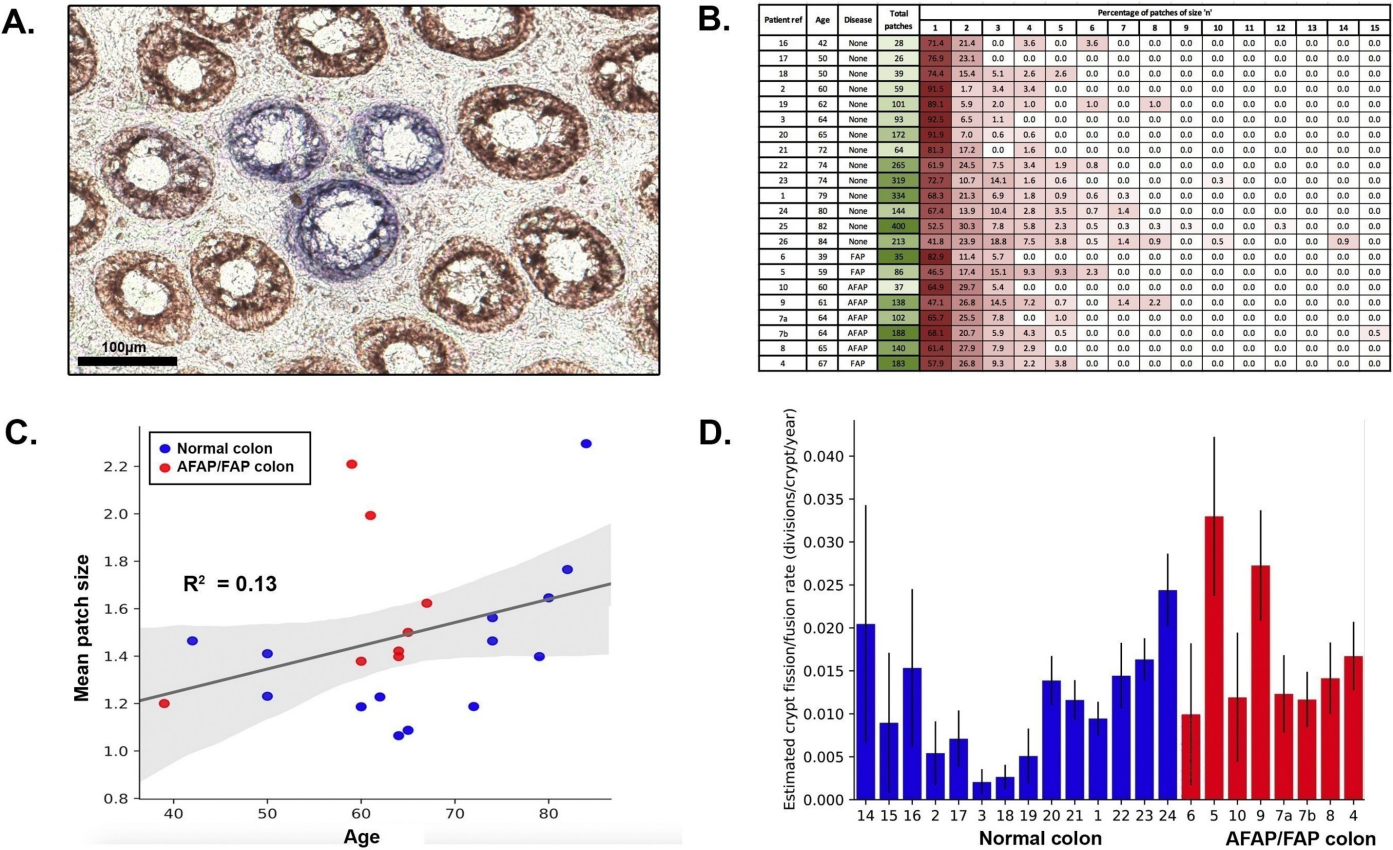
Analysis of cytochrome c oxidase (CCO) patch size distribution. (A) Representative example of a CCO-deficient patch of three crypts in the colonic epithelium. Scale bar represents 100 μm. (B) Distribution of total CCO-deficient patches, and number of patches of size n for each patient. (C) Relationship between patient age and mean CCO-deficient patch size. Shown is the age and mean CCO-deficient patch size of each individual patient (blue dots represent the disease-free ‘normal’ colon, and red dots represent the attenuated familial adenomatous polyposis [AFAP]/familial adenomatous polyposis [FAP] colon). The black line represents the predicted mean patch size over time for a hypothetical patient with a fission and fusion rate equal to the mean fission and fusion rate of the patient cohort. (D) Estimated fission/fusion rate for each patient, arranged first by disease status, then by ascending age. Error bars represent 95% CIs.

We next sought to estimate the rate of crypt fission and fusion in the human colon, defined as the number of fission or fusion events an individual crypt undergoes per year. For this analysis, we used only the distribution of CCO− crypt ‘patch size’—where a patch was defined by the number of adjacent CCO− crypts (figure 2A). Therefore, we were able to analyse a larger cohort of 21 patients, in each of which we measured CCO− patch size in an area representing at least 2000 total crypts (14 disease-free patients and 7 AFAP/FAP patients, figure 2B and C, patients 1–10 and 14–24 in table 1, data largely from Baker *et al* 3). Previous estimates of the crypt fission rate derived from this type of data have not considered the impact of crypt fusion.^3 10^ We reasoned that fission of a CCO− crypt would increase the patch size by 1, whereas fusion would decrease the patch size by 1 if two CCO− crypts fused, or decrease the patch size by 1 with 50% probability if a CCO− and a neighbouring CCO+ crypt fused. We used a birth-death process to model CCO− patch size evolution (see online supplementary mathematical note).

Mathematically, the fission and fusion rates are not separately identifiable from the patch size distribution alone (see online supplementary mathematical note). Consequently, given that we found evidence in support of balanced rates of fusion and fission, we set their respective rates to be equal. For simplicity, we considered fusion and fission events to be spatially and temporarily uncorrelated so that the average crypt number is constant, while the local density may fluctuate (see below for an assessment of this assumption). Then, based on the predicted distribution of patch sizes, a maximum likelihood estimate of the fission rate was made for each sample (table 1, figure 2D, online supplementary figure 3), with a mean fission rate in the disease-free colon of 0.011 divisions/crypt/year (range 0.002–0.024), corresponding to a mean crypt cycle length of approximately 90 years. This estimate is approximately 20% higher than if only fission were simulated (mean fission rate=0.009 divisions/crypt/year [range 0.002–0.018], crypt cycle=110 years). A previous investigation of the fission rate that did not consider the potential for crypt fusion and was based on histological analysis of an alternative neutral marker was 0.0068 divisions/crypt/year, translating to a crypt cycle of approximately 150 years.^10^ One potential explanation for the small difference between these two rate estimates is divergence in the age-related accumulation of the differing clonal marks used in the respective studies, due to unmeasured fluctuations in the mutation rate (labelling rate) or non-neutral evolutionary dynamics of the mutant clones.

Similarly, the crypt fission rate in the AFAP/FAP colon was inferred from CCO patch size distributions. The mean fission rate of these patients was 0.017 divisions/crypt/year (range 0.0099–0.032, table 1, figure 2D), corresponding to a crypt cycle length of 60 years. This was not significantly different to that of the disease-free colon (p=0.11 by the two-sided Mann-Whitney U test), but we acknowledge that the sample size of our study means that it was not powered to detect smaller differences in fission/fusion rates.

### Estimation of the duration of fission/fusion

In mouse, the duration of crypt fission is approximately 1 week.^13^ The duration of fission in human samples is unknown as longitudinal measurements are not feasible. We used the per-patient estimate of the crypt fission rate and the number of bifurcating crypts in a single section to estimate the time taken to complete fission. We assumed that approximately half of observed bifurcations were the result of fission. Thus, the duration of crypt fission was mathematically estimated as the fraction of crypts undergoing fission at a given time divided by the fission rate. The median fission duration for the 10 informative patients (3 disease-free and 7 AFAP/FAP, patients 1–10 in table 1) was calculated to be 9 weeks (range 4–41 weeks, table 1). There was no statistically significant difference between fission duration in the healthy colon and the AFAP/FAP colon (p=0.18 by the two-sided Mann-Whitney U test).

### Spatial correlation of fission and fusion events

Local variations in the microenvironment could conceivably drive crypt fission/fusion events. To examine this hypothesis, we examined whether bifurcation events were spatially correlated. We selected samples consisting of at least 2000 crypts (three disease-free patients and seven AFAP/FAP patients), and computed the Ripley’s L spatial clustering statistic on the locations of bifurcating crypts. We found that six samples (samples 4, 6, 7a, 7b, 8 and 10) showed statistically significant spatial correlation of bifurcation events (p<0.01; Monte Carlo test; online supplementary figure 4 and online supplementary mathematical note). The low numbers of type II and type III crypts precluded formal assessment of clustering of putative fission and fusion events.

We note that if fission/fusion acts to maintain homeostasis of local crypt density by respectively increasing/decreasing local crypt number, then a reasonable hypothesis is that when averaged over long times fission/fusion rates are fairly uniform across the colon. Indeed, the good fit of our model with uniform fission/fusion rates, and lack of multimodality in the patch size data (online supplementary figure 3) is supportive of this idea.

### Methylation analysis in a bifurcating crypt provides support for fusion

**Figure 3:**
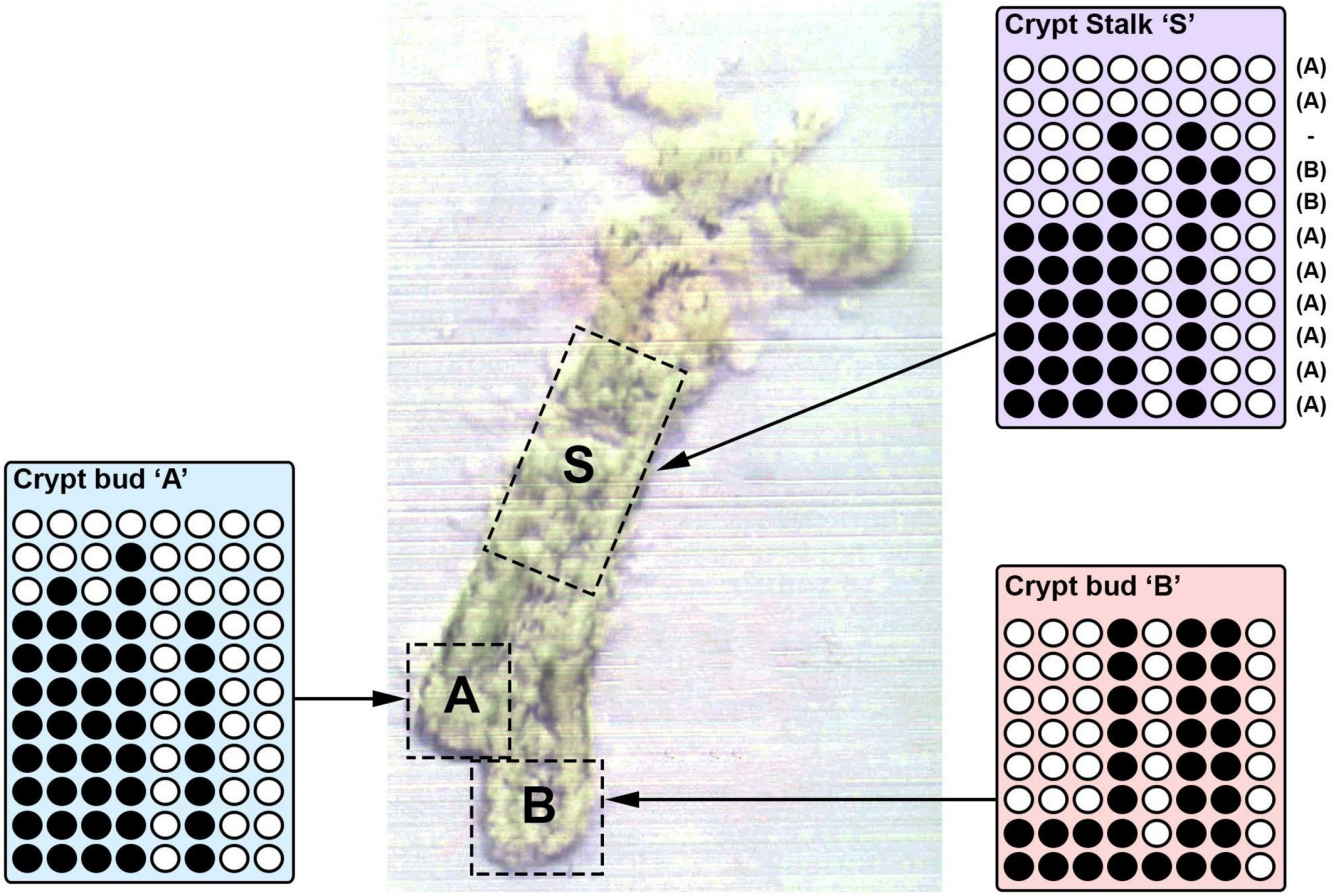
Analysis of cardiac-specific homeobox (*CSX*) methylation in a bifurcating crypt. Image of a bifurcating crypt, with the two buds (‘A’ and ‘B’) and the crypt stalk ‘S’ isolated and used for analysis of *CSX* methylation status. Each row represents the *CSX* methylation tag of an individual clone. Open circles represent an unmethylated CpG site and closed circles represent a methylated CpG site. The two buds share no methylation tags, and the stalk contains tags from both ‘A’ and ‘B’.

Finally, we sought to provide evidence of crypt fusion using an orthogonal methodology. DNA methylation at CpG sites in unexpressed genes is subject to neutral drift, and so closely related crypts are expected to have more similar methylation tags than unrelated crypts.^18^ We and others have previously used bisulfite sequencing to analyse the methylation patterns of both arms of branching crypts (presumed fission intermediates) and unexpectedly found them to be as distinct as unrelated crypts.^18 19^ We hypothesised that an explanation of this observation is that branched crypts could be intermediates of fusion rather than fission. In a single bifurcating crypt isolated from a patient with IBD, we performed bisulfite sequencing of the cardiac-specific homeobox (*CSX*) gene (unexpressed in the human colon) in the two arms and the stalk of the bifurcating crypt (figure 3). We found that the two arms did not share any methylation tags, suggesting they are not closely related. Furthermore, we found that the stalk contained a mixture of tags found in both arms. This ‘perfect segregation’ of methylation tags in the two arms of the bifurcating crypt is suggestive of a fusion event.

## Discussion

Here, we provide evidence that the merging of adjacent colon crypts—the process of crypt fusion—occurs in the human colon. Our analysis is suggestive that, in healthy bowel, the rate of crypt fusion matches the rate of crypt fission, suggesting that fission/fusion events are homeostatic mechanisms that together maintain the population size of crypts in the colon. While our analysis does not rule out the potential further mechanisms of crypt death (such as ‘crypt apoptosis’), the evidence provided herein, that fission/fusion occur at approximately equal rates does suggest that alternative mechanisms of crypt loss, such as death, are likely to be rare events. In general, that crypts are capable of fusing further highlights the plasticity of the epithelial cell population in the colon. We and others have thought of crypts/glands as the basic ‘units of selection’ throughout the GI tract^20^—in other words that crypts can be considered as automatous homogeneous units to explain patterns of intercrypt clonal expansion in the colon. That crypts can fuse—and so merge their identity—requires nuancing of this simplistic view.

The cellular-molecular mechanisms that regulate crypt fission and fusion remain to be elucidated. Potentially, fusion could be induced to ‘relieve’ local mechanocellular stresses induced by a prior fission of a nearby crypt. Indeed, we observe spatial clustering of bifurcation events (online supplementary figure 4). Of much interest is the dynamics of niche-producing stromal cells^21 22^ from each crypt—prior to a fusion event there are two ‘sets’ of these cells that reduce to a single set. Understanding how this is achieved may yield further mechanistic insight into the maintenance of homeostasis in the gut. Resolution of the induced stresses and strains in the basement membrane during the merger is also of interest. Similarly, determination of how epithelial cell fate is regulated during the merger process—particularly at the ‘saddle point’ between the two merging crypts—could be important for furthering our understanding of epithelial cell regulation in the crypt. Furthermore, if and how the dynamics of fission/fusion change during ageing and along the length of the colon may hold clues to the development and maintenance of the rapid-renewing intestinal epithelium throughout life.

The evolutionary pressure for crypt fission/fusion turnover is unclear. It is conceivable that fusion could be a competitive process, whereby less ‘fit’ crypts are replaced by a fitter neighbour: in this regard, fusion could be way to heal ‘sick crypts’ (presumably less fit) without compromising epithelial barrier integrity. A fanciful idea is that crypt fusion could be tumour-suppressive via engulfment, and subsequent removal, of a ‘cancerised’ crypt^23^ by a healthy neighbour. On the other hand, such a mechanism could be subverted and drive the expansion of the mutant clone. Of course, some of kind of sensing between crypts—either direct or indirect—needs to be present for this type of crypt-competitive mechanism to be plausible. Certainly, as has been previously noted, fusion of a ‘cancerised’ crypt with a wild-type crypt would restart stem cell competition of a previously ‘fixed’ lineage, and so could lead to removal of tumourigenic mutations via a passive means.^13^ That fusion is simply a chance process, driven by stochastic events in the positioning of crypts and the epithelial cells within, the position and status of supporting microenvironmental cells, and/or the integrity of the basement membrane, also cannot be ruled out. Furthermore, we recognise the possibility that due to intrinsic or microenvironmental factors, fission or fusion events may not necessarily always reach completion, but may stall or even reverse. Although it is not possible to observe such ‘stalled’ events using our analysis, this eventuality could be more readily examined in mouse models where longitudinal observation is possible. If fission/fusion can reverse it would imply that the crypt life cycle is a highly fluid and adaptable, and further reject the notion of the crypt as a ‘clonal unit’.

Mutations that occur within the stem cells of a crypt can spread throughout the crypt via a process of neutral competition. Recent estimates of the clonal fixation time within a crypt by Nicholson *et al* 10 (median 6.3 years) are significantly longer than previous estimates from human data^3^ and experimental data from mouse models.^6 7^ Crypt fusion provides a mechanism, whereby crypt ‘polyclonality’ can be reintroduced into a crypt, via the merging of two differentially labelled crypts. Accordingly, fusion would inflate the number of partially fixed crypts (compared with the case without crypt fusion), possibly leading to an underestimate of the effective replacement rate, or equivalently, an overestimate of the fixation time. Accurate measurement of the distribution of clone sizes within partially labelled crypts is a route to decouple neutral drift dynamics from the effects of fusion, but such data have not yet currently been generated.

Our mathematical analysis relies on a number of assumptions, foremost that stem cell numbers per crypt are small and constant, and that crypt fission causes equal segregation of stem cells between the two arms of the bifurcating crypt. We also assume that CCO deficiency is a neutral mark, that is induced at a constant rate throughout life. Although we consider that these assumptions are reasonable, we note that relaxing them weakens the strength of our evidence for crypt fusion. Nonetheless, we maintain that crypt fusion provides a parsimonious explanation of the high number of ‘type III’ bifurcation events that we have observed. In our estimation of the rate of crypt fusion, we note that we have neglected to consider other mechanisms of ‘crypt extinction’, and inclusion would alter our estimate of the crypt fission/fusion rate. The strength of our conclusions are naturally limited by the available sample size, and the corresponding accuracy of the inferred fission/fusion rates are reflected by the reported broad CIs.

In summary, we present evidence of crypt fusion as a homeostatic process in the human colon, nuancing our view of growth regulation in this rapidly renewing epithelium.

## Methods

### CCO staining

Two-colour enzyme histochemistry for CCO activity was performed on serial sections at 12 µm thickness as previously described.^11^

### Analysis of CCO activity, bifurcation events and CCO patch size

A representative CCO-stained section was selected for each sample, and this section was used for manual quantification of CCO activity and bifurcation events. We recorded the number of wild-type crypts (brown stain) and the number of crypts deficient of CCO activity (blue). Crypts with a fraction of the crypt that was CCO-deficient were designated ‘partial crypts’.

Bifurcation events were identified as ‘8-shaped’ pairs of crypts that shared a portion of their border, and each event was verified as a true bifurcation event by following the crypts through serial sections. Each event was classified as ‘type I’ (involving two CCO-proficient crypts), ‘type II’ (involving two CCO-deficient crypts) or ‘type III’ (involving a CCO-proficient crypt and a CCO-deficient crypt). Rare bifurcation events that involved partial CCO-deficient crypts were classed as ‘type I’ events.

### Three-dimensional reconstruction of crypt bifurcation

A fresh frozen sample of colonic epithelium from patient 7 was sectioned through at 6 µm thickness and stained for CCO activity as described above. Individual sections were scanned and registered in FreeD for 3D reconstruction, as follows. Serial images (TIFF file format) were imported into FreeD software V.1.10 image stack files. Gland boundaries were drawn manually in each two-dimensional (2D) serial image and connected along the third dimension between adjacent slides. This procedure is facilitated by simultaneous display of masked gland boundaries during virtual microscopy in FreeD software. After manual 2D assessment of all virtual tissue slides of one stack, 3D models can be computed and visualised by interconnection of the defined masks along the third dimension in FreeD software.

### Crypt isolation

A fresh tissue biopsy from a female aged 66 years with inactive IBD was sampled immediately after endoscopic removal. The biopsy tissue was incubated at 37°C for 10 min in calcium-free and magnesium-free Dulbecco’s modified eagle medium (DMEM) containing 30 mmol/L EDTA. The tissue was then agitated in DMEM containing calcium and magnesium for 30 s to separate the crypts from the lamina propria mucosa and fibrous stroma. A bifurcating crypt was observed, photographed and isolated under a dissecting microscope (Olympus model SZ60). The two arms and the stalk were separated and used for methylation analysis.

### Methylation analysis

Analysis of methylation patterns was performed as previously described.^19^ Briefly, DNA was extracted from the isolated crypt stalk and arms using the Arcturus Picopure DNA extraction buffer (Thermo Fisher Scientific) then bisulfite converted. A nested PCR was used to amplify the non-expressed *CSX* locus. PCR products were single-strand cloned, sequenced and analysed for methylation of the 8 CpG islands in the locus. The pattern of methylation revealed in each DNA strand was termed a ‘tag’.

10.1136/gutjnl-2018-317540.supp2 Supplementary data

10.1136/gutjnl-2018-317540.supp3 Supplementary data
