# Supplementary material for: Crypt fusion as a homeostatic mechanism in the human colon
Source: Gut. 2019 Mar 14;68(11):1986–93. doi: 10.1136/gutjnl-2018-317540 (PMC6839731; doi:10.1136/gutjnl-2018-317540)
Supplement: Supplementary data [file gutjnl-2018-317540supp001.pdf]

Supplementary Mathematical note

Evaluation of the null hypothesis that crypt fission leads to the observed proportion of perfectly segregated bifurcating crypts

We assumed that crypt fission is 'symmetric' such that both daughter crypts inherit exactly S/2 of the total number of stem cells S in the parent crypt. Then only crypts with exact half of the stem cells labelled can potentially divide to produce a perfectly segregated bifurcating crypt (type III bifurcation). The case of an odd number of stem cells is considered separately below. The probability of a crypt undergoing fission giving rise to perfectly segregated arms can be estimated as:

$$p = \frac{1}{B} \times \frac{\mathrm{N_{half}}}{\mathrm{N_{total}}}$$

Where B is the number of possible bifurcation planes, which we assumed was simply equal to half of the number of stem cells (S/2), N_{half} is the number of crypts that have S/2 CCO- stem cells and N_{total} is the total number of counted crypts. Due to the difficulty in counting the number of crypts which have exactly half their stem cells labelled, N_{half} was estimated by multiplying the measured number of partially labelled crypts ($N_{partial}$) by the expected proportion of partially labelled crypts that have half of their cells labelled. The probability distribution of clone sizes within partially labelled crypts under the assumption of neutral genetic drift at long times[1] is given by:

$$P(n\ labelled\ stem\ cells\ in\ crypt) = \frac{2(S-n)}{S(S-1)}$$

Hence, we can estimate N_{half} as:

$$N_{half} = \frac{\mathrm{N_{partial}}}{S-1}$$

allowing us to estimate p as:

$$p = \frac{2}{S(S-1)} \frac{\mathrm{N_{partial}}}{\mathrm{N_{total}}}$$

Therefore, under the null hypothesis, the probability of 21 (N_{seg}) or more of the 309 bifurcating crypts ($N_{bifurcate}$) to be perfectly segregated is then given by the binomial distribution:

$$P(\mathrm{N_{seg}}) = 1 - \sum_{k=0}^{N_{seg}-1} \binom{\mathrm{N_{bifurcate}}}{k} p^k (1-p)^{\mathrm{N_{bifurcate}}-k}$$

Probability of perfect segregation in a crypt with an odd number of stem cells

Above, the probability of a crypt undergoing fission perfectly segregating all CCO- and CCO+ cells to each arm of the bifurcating crypt was discussed for the case of an even number of stem cells. Considering the case where the number of stem cells is odd is straightforward. Instead of each arm of the crypt receiving S/2 stem cells, one arm receives (S+1)/2 and the other (S-1)/2. The number of possible bifurcation planes is then S. In analogue to the even case, the probability of a crypt undergoing fission giving rise to perfectly segregated arms can be estimated as:

$$p = \frac{1}{B} \times \left(\frac{N_{\frac{S+1}{2}} + N_{\frac{S-1}{2}}}{\mathrm{N_{total}}} \right)$$

Substituting the distribution of partially labelled crypts as described above, we estimate p to be the same as in the even case:

$$p = \frac{2}{S(S-1)} \frac{\mathrm{N_{partial}}}{\mathrm{N_{total}}}$$

The rest of the calculation proceeds as previously.

Crypt fission and fusion rate

Crypt fission allows the expansion of patches of clonal tissue within the colon. Previous studies[2, 3] have attempted to infer the rate of crypt fission from the distribution of patch sizes, identifying clonal patches using neutral mutational markers (such as CCO deficiency, as used in this study). These studies assumed that clonal patches can only grow via crypt fission, however the existence of crypt fusion suggests that clonal patches can also shrink.

We modelled the processes of crypt fission and fusion as a linear birth-death process. The rate at which a patch transitions from containing m labelled crypts to containing $m+1$ labelled crypts is the fission rate per crypt, $\kappa_{fission}$, multiplied by the patch size m. The death rate is more complicated to calculate. If a CCO- crypt fuses with a neighbouring CCO+, the patch size will decrease by 1 half of the time (e.g. for the 50% of cases where the CCO+ clone 'wins'). If instead a CCO- crypt fuses with another CCO- crypt within the patch, the patch size will always decrease by 1. The rate at which a patch decreases in size depends on the size of the patch. For simplicity we shall consider the case where the crypts are organised into a regular structure with 4 nearest neighbours, in line with the observed average coordination in the colon. An isolated CCO- crypt has 4 unlabelled neighbours, so that loss will occur at rate $\frac{\kappa_{fusion}}{2}$. Each CCO- crypt in a patch of size 2 has 3 unlabelled neighbours and 1 labelled neighbour, so each crypt will transition to CCO+ at a rate $\frac{\kappa_{fusion}}{4} + \frac{3\kappa_{fusion}}{4} \times \frac{1}{2} = \frac{5\kappa_{fusion}}{8}$. In this manner, the rate at which larger patches shrink can be calculated relatively simply. However, 95% of the patches observed contained 4 or fewer crypts. Therefore, to allow the application of an analytically-tractable linear birth-death model, we calculate an effective crypt loss rate by finding the weighted mean of the loss rate for patches of size 4 or less. For our data, we find an effective loss rate of 0.544 κ_{fusion}, hence we model the rate at which a patch of size m transitions to a patch of size $m-1$ as 0.544 $\kappa_{fusion} m$.

We know the rate at which crypts transition from wild type (CCO+) to fully fixed (CCO-) is $\mu \mathrm{N_{total}}$ where μ is the rate at which clonally converted CCO- crypts are spontaneous formed per unit time and $\mathrm{N_{total}}$ is the total number of crypts assessed. We assume $\mathrm{N_{total}}$ is sufficiency large to remain approximately constant despite crypt labelling. Thus, the crypt 'birth' rate $B = \kappa_{fission}$ and 'death' rate is $D \approx 0.544\ \kappa_{fusion}$. The differential equations describing the patch size distribution are (where K_m is the probability of seeing a patch of size m at time t):

For $m = 1$:

$$\frac{dK_1}{dt} = -(B+D)K_1 + 2DK_2 + \mu \mathrm{N_{total}}$$

$m \geq 2$:

$$\frac{dK_m}{dt} = -(B+D)mK_m + (m-1)BK_{m-1} + (m+1)DK_{m+1}$$

This is a complicated equation to solve directly, but we can consider the case of a single birth-death process that starts at $m = 1$, for which solutions are known, and then integrate the resulting distribution over time to find the time dependent patch size distribution. For a single birth-death process, the differential equations are:

$$\frac{dp_m}{dt} = -(B+D)mp_m + (m-1)Bp_{m-1} + (m+1)Dp_{m+1}$$

We define the simplifying functions:

$$\alpha = \frac{D\left(e^{(B-D)t} - 1\right)}{Be^{(B-D)t} - D}$$

$$\beta = \frac{B\left(e^{(B-D)t} - 1\right)}{Be^{(B-D)t} - D}$$

If we have the initial condition $p_a(t=0) = \delta_{a1}$, then the solution is[4]:

$B \neq D \wedge m \geq 0$:

$$p_m(t) = \beta^{m-1}(1-\beta)(1-\alpha)$$

$B \neq D \wedge m = 0$:

$$p_0(t) = \alpha$$

$B = D \wedge m \geq 0$:

$$p_m(t) = \frac{(Bt)^{m-1}}{(1+Bt)^{m+1}}$$

$B = D \wedge m = 0$:

$$p_0(t) = \frac{Bt}{1+Bt}$$

The integrated crypt size distribution can then be calculated as $K_m(t) = \frac{1}{T}\int_0^T p_m(t')\,dt'$, leading to the following equations:

$B \neq D \wedge m \geq 1$:

$$K_m(T) = \frac{\beta^m}{BmT}$$

$B \neq D \wedge m = 0$:

$$K_0(T) = 1 - \frac{1}{BT}\log\left(\frac{1}{1-\beta}\right)$$

$B = D \wedge m \geq 1$:

$$K_m(t) = \frac{1}{BmT}\left(\frac{BT}{1+BT}\right)^m$$

$B = D \wedge m = 0$:

$$K_0(T) = 1 - \frac{1}{BT}\log(1 + BT)$$

However, we can only measure surviving CCO- crypts. Hence, we must renormalize the crypt size distribution to that of the "persisting" patches, $K'_m(t) = \frac{K_m(t)}{1-K_0(t)}$.

$B \neq D \wedge m \geq 1$:

$$K'_m(T) = \frac{\beta^m}{m\ \log\left(\frac{1}{1-\beta}\right)}$$

$B = D \wedge m \geq 1$:

$$K'_m(t) = \frac{1}{m\log(1 + BT)}\left(\frac{BT}{1 + BT}\right)^m$$

Prior to fitting the patch size distributions, we corrected our data for the possibility of spontaneous, independent CCO- mutations in adjacent crypts, leading to the misclassification of two individual clones as a patch of size 2. If we estimate the probability that a crypt becomes labelled, $p = \mu T$, as the number of labelled patches divided by the total number of crypts, we can estimate the number of patches of size 2 that are, in fact, separate clones as $2p^2(1-p)^6 \mathrm{N_{total}}$ (where we are still assuming that the crypts are organized into a regular structure with 4 nearest neighbours). Similar corrections could be performed for patches of size 3 and above, however $p \ll 1$ so the number of misclassified size 3 patches was assumed to be negligible.

To fit to the data, we performed a maximum likelihood estimate on a patient-by-patient basis (Supplementary Figure 2). The log-likelihood of observing $N_{patches}$ patches, each of size m_i, is:

$$\mathcal{L}(b, d|\mathrm{m_i}) = \sum_{i=1}^{\mathrm{N_{patches}}} \mathrm{m_i}\log\beta - \log\left(\mathrm{m_i}\log\left(\frac{1}{1-\beta}\right)\right)$$

The log-likelihood is purely a function of β, and $\beta(B, D)$ is a many-to-one function, hence we cannot separately determine $\kappa_{fission}$ and κ_{fusion} from our data. Instead, the fission rate was fixed to be equal to the fusion rate, as discussed in the main text. The log-likelihood was maximised numerically using the 'Nelder-Mead' method and the confidence intervals on each fit were estimated from the Fisher information matrix.

The duration of crypt fission/fusion

If we assume that the rate of crypt fission per crypt is constant over time, then the probability that a given crypt undergoes fission in a time-interval Δt is simply $\kappa_{fission}\Delta t$. Let the duration of crypt fission be T, then if we observe a crypt undergoing fission at a given time t, that bifurcation must have begun later than $t - T$. Hence, the probability that a crypt is undergoing crypt fission at a given time is $\kappa_{fission}T$. Due to the inability to accurately evaluate Type I bifurcations as fission or fusion events, we must estimate the number of bifurcations arising from crypt fission as half the total number of bifurcations ($N_{bifurcate}$). If we model the number of crypt-fission bifurcations as a binomial process with probability $\kappa_{fission}T$, then we can estimate the duration of crypt fission as $T = \frac{N_{bifurcate}}{2\kappa_{fission}N_{total}}$.

The spatial distribution of bifurcation events

Visual examination of the positions of fission/fusion events in the sample (Supplementary Figure 4) indicated spatial clustering of events. To test this hypothesis, the positions of each bifurcation event within a sample were extracted, along with the region of the image (R) containing colon tissue.

Ripley's L function (a variance stabilised transformation of Ripley's K function) is a descriptive statistical measure of spatial homogeneity. Comparing Ripley's L function for a given spatial distribution of points to 999 (N_{sim}) Monte Carlo simulations of randomly distributed points allows the presence of clustering to be inferred. Ripley's L function at a search radius, r, is given by:

$$\hat{L}(r) = \sqrt{\frac{1}{\pi\hat{\lambda}}\sum_{i=1}^{n}\sum_{j\neq i}^{n}\frac{H(r-d_{ij})}{nw_{ij}}}$$

Where d_{ij} is the pairwise distance between the ith and j^{th} points, λ is the mean density of points (estimated as $\hat{\lambda} = \frac{n}{A}$), r is a search radius defining the scale of spatial interactions, H is a Heaviside step function and w_{ij} is an edge correction factor (w_{ij} is the fraction of the circumference of a circle of radius d_{ij} centred on the point that lies within the sampling region. For a spatially homogeneous Poisson process $\hat{L}(r)$ should approximately equal r. To determine whether deviations from r are due to spatial clustering or consistent with random noise, a set of 999 simulations containing n randomly scattered points within R were generated, and for each simulation Ripley's L function ($L_{sim}(r)$) was calculated. For each simulation, the maximum difference between the simulation and the theoretical value of L, $D = \max(|L_{sim}(r) - r|)$, was recorded. To construct a global simulation envelope of significance $\alpha = 0.01$, the 10th largest D value was found. If any value of $\hat{L}(r)$ has a larger deviation from r than this critical value, then the spatial distribution is significantly different from a spatially homogeneous Poisson process.

For a significance of 0.05, samples 4, 6, 7a, 7b, 8 and 10 exhibited evidence of spatial clustering (Supplementary Figure 4). This analysis is robust to our choice of α, for a stringent significance of $\alpha = 0.001$, only sample 7b is no longer statistically significant. Note that all of these patients are FAP or AFAP, and each sample contained far larger numbers of bifurcations than the IBD or disease-free samples. This analysis cannot distinguish between underlying spatial heterogeneity of the fission/fusion rates within the sample, or local interactions between fission/fusion events.

References (Supplementary Mathematical note)

1. Simons BD. Deep sequencing as a probe of normal stem cell fate and preneoplasia in human epidermis. Proc Natl Acad Sci U S A 2016;113:128-33.
2. Baker AM, Cereser B, Melton S, et al. Quantification of crypt and stem cell evolution in the normal and neoplastic human colon. Cell Rep 2014;8:940-7.
3. Nicholson AM, Olpe C, Hoyle A, et al. Fixation and Spread of Somatic Mutations in Adult Human Colonic Epithelium. Cell Stem Cell 2018;22:909-918 e8.
4. Bailey NTJ. The Elements of Stochastic Processes with Applications to the Natural Sciences. Mathematics of Computation 1990;19:153.

Baker *et al*, Supplementary Figure 1

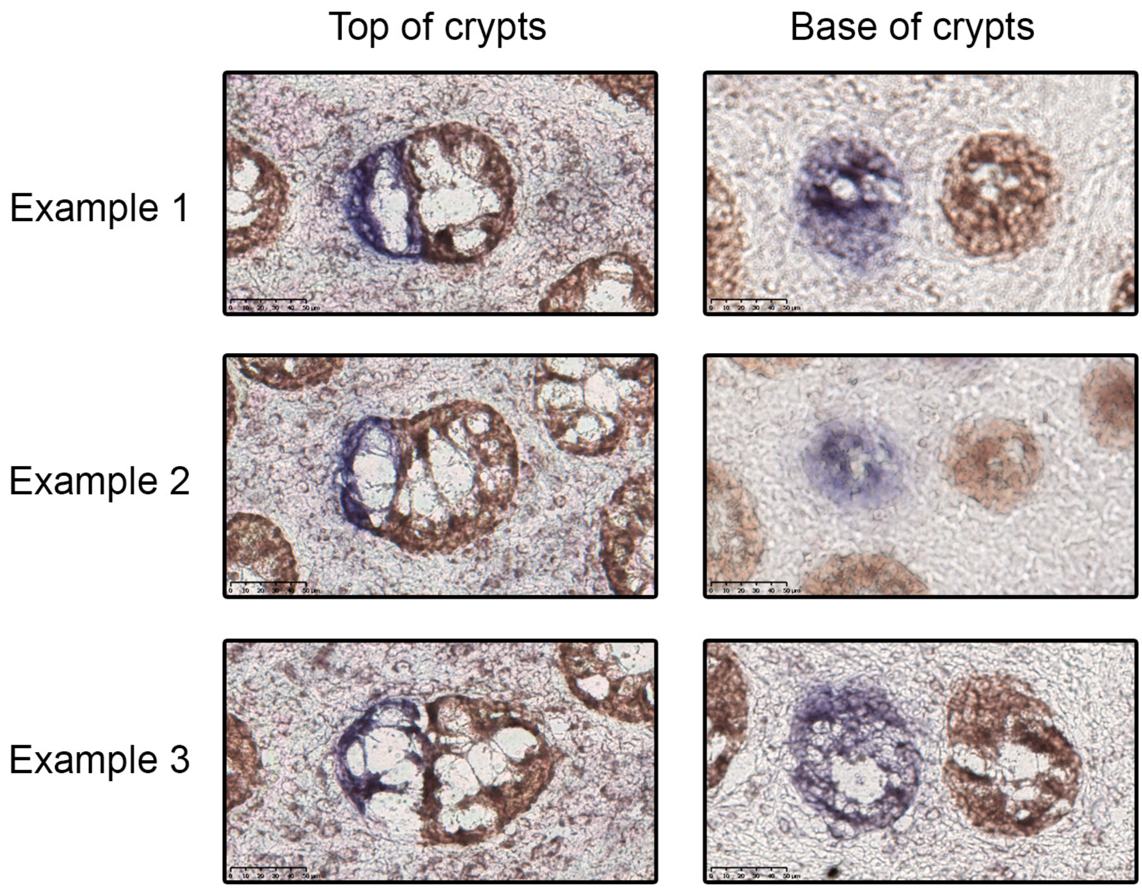

Baker *et al*, Supplementary Figure 2

A.

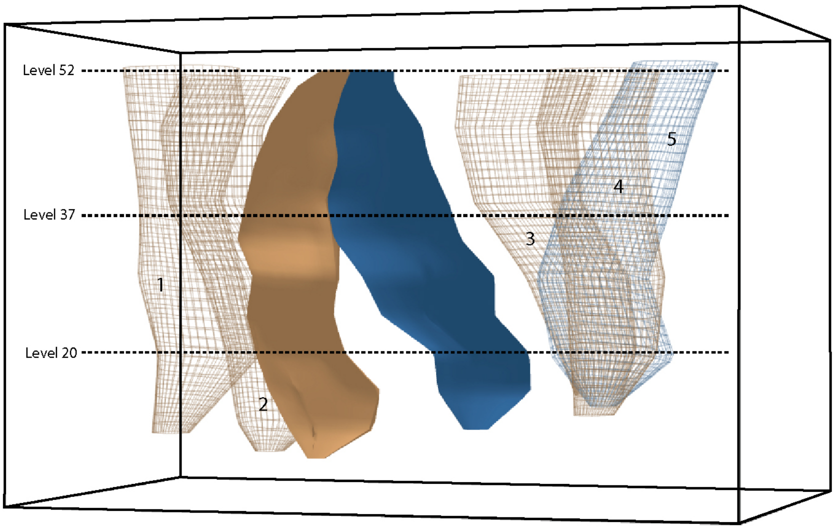

B.

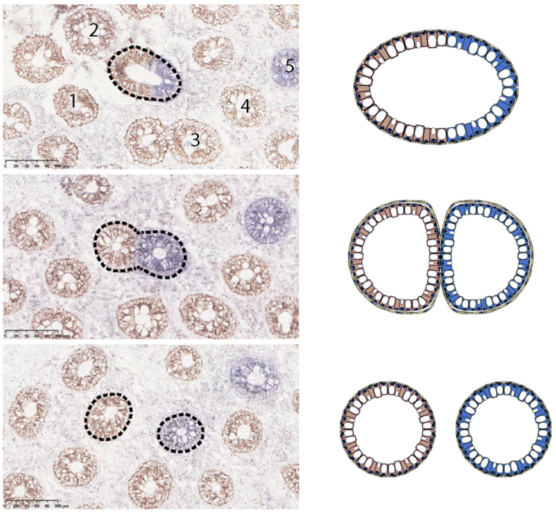

Baker *et al*, Supplementary Figure 3

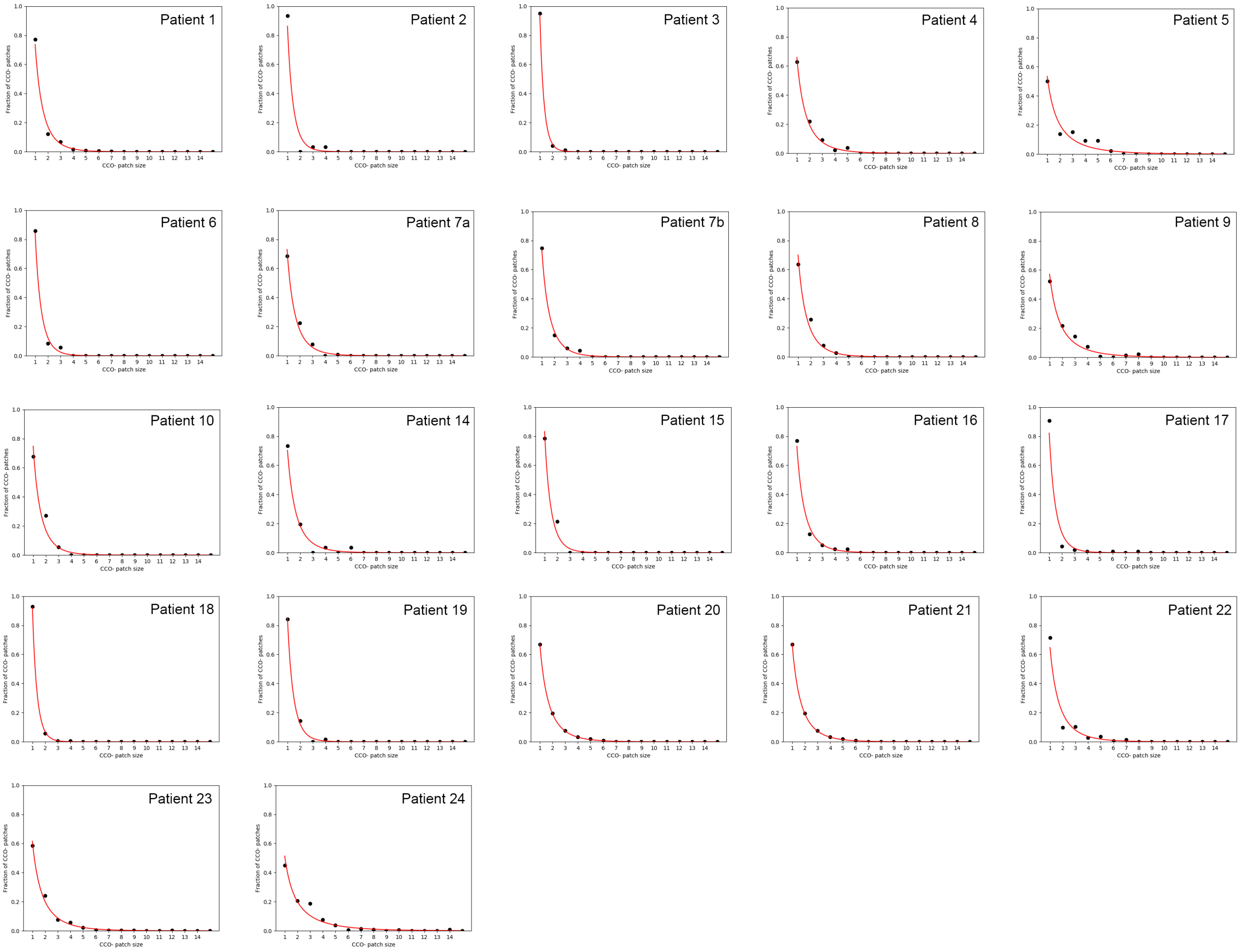

Baker *et al*, Supplementary Figure 4

Sample 1

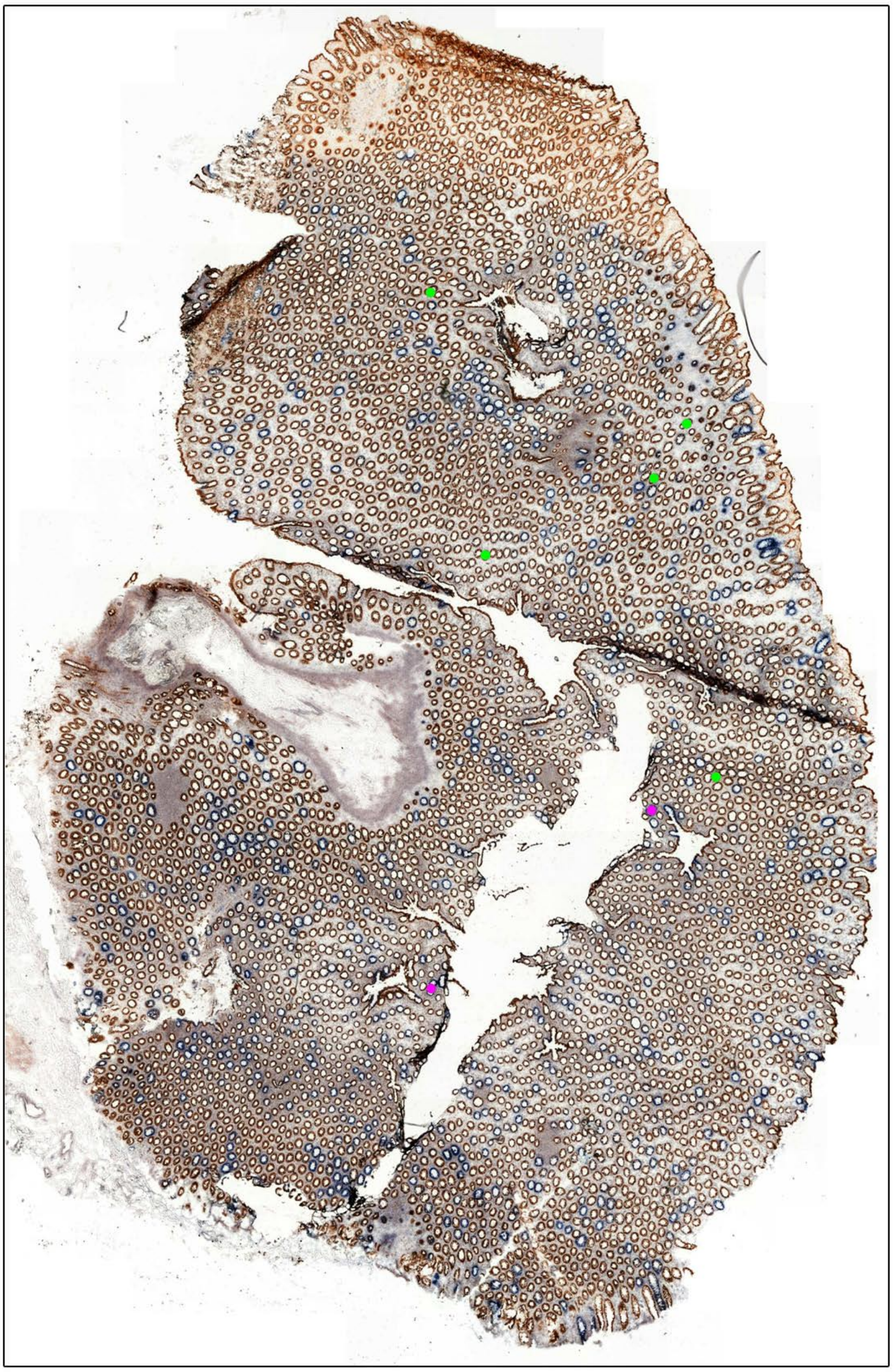

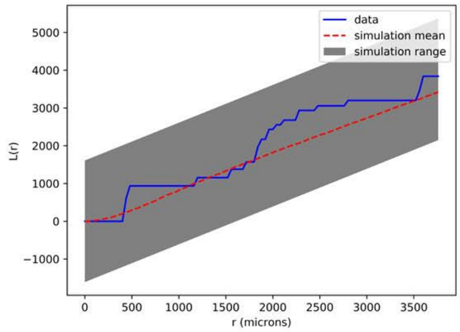

data
simulation mean
simulation range
5000
4000
3000
2000
1000
0
−1000
L(r)
0
500
1000
1500
2000
2500
3000
3500
r (microns)

Sample 2

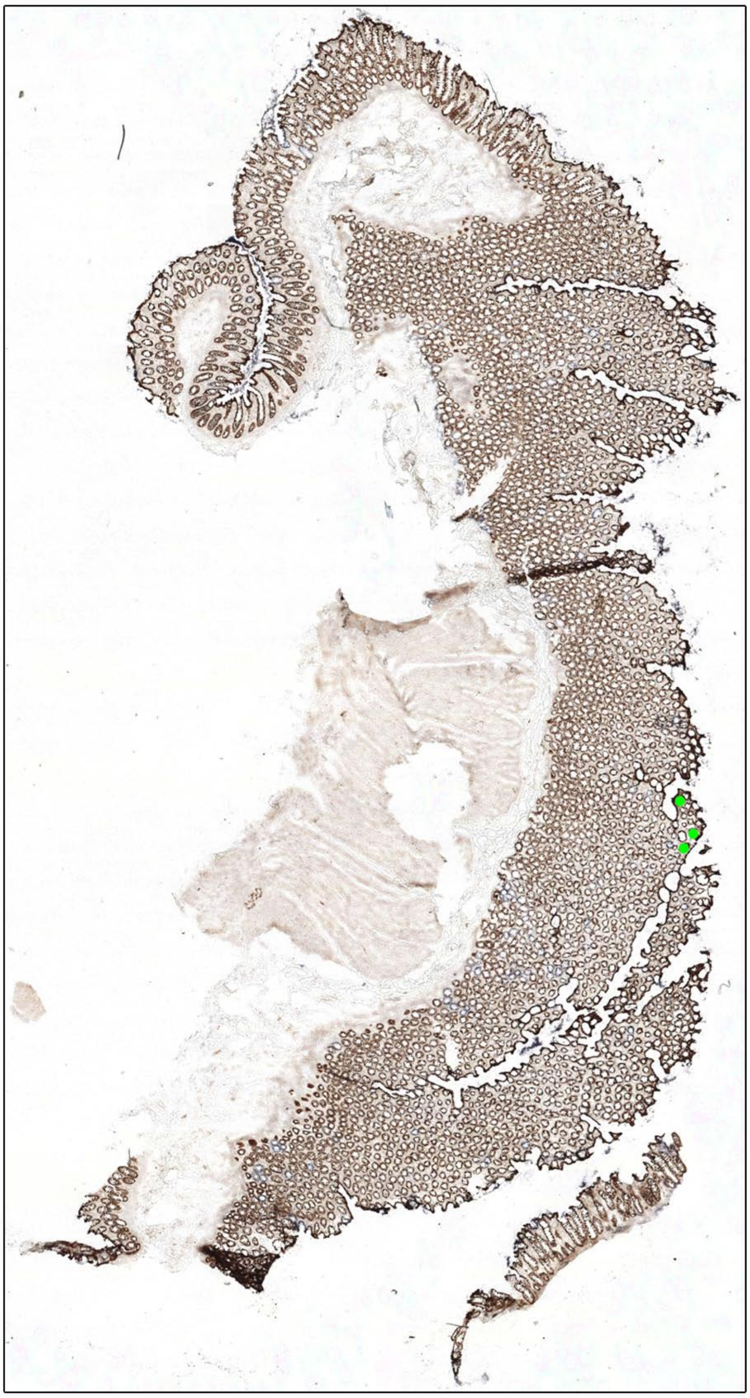

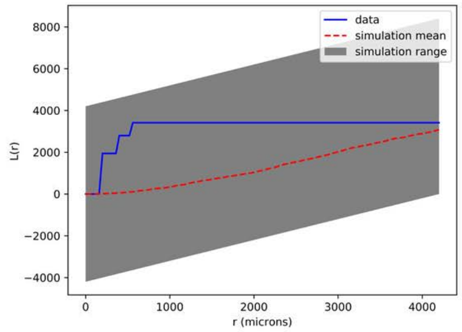
data
simulation mean
simulation range
8000
6000
4000
2000
0
−2000
−4000
L(r)
0
1000
2000
3000
4000
r (microns)

Sample 3

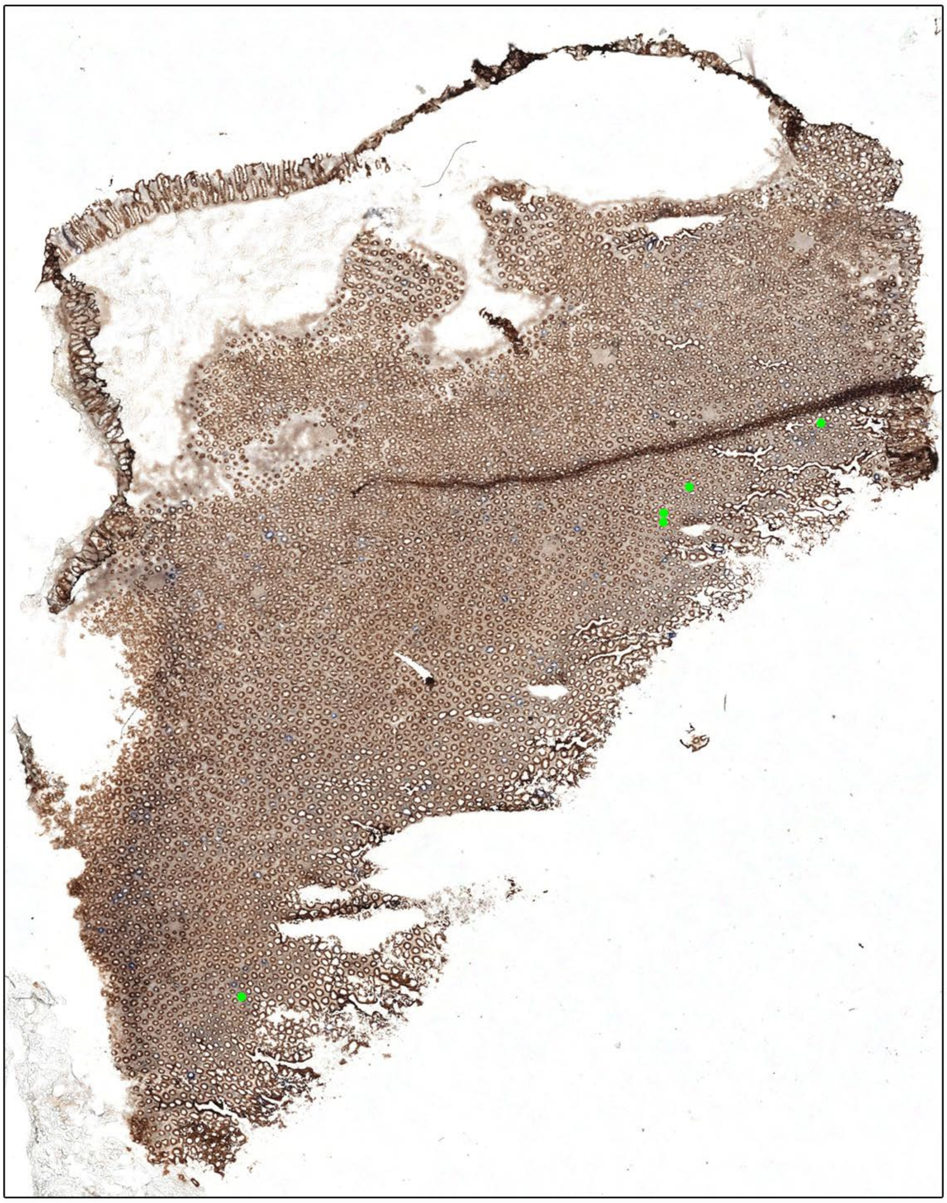

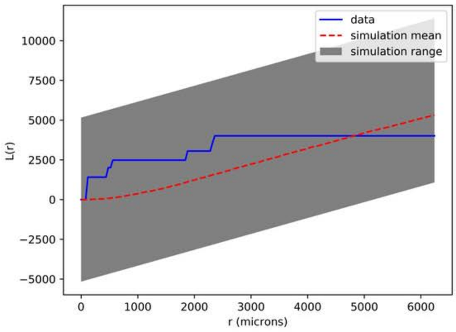
data
simulation mean
simulation range
10000
7500
5000
2500
0
−2500
−5000
L(r)
0
1000
2000
3000
4000
5000
6000
r (microns)

Sample 4

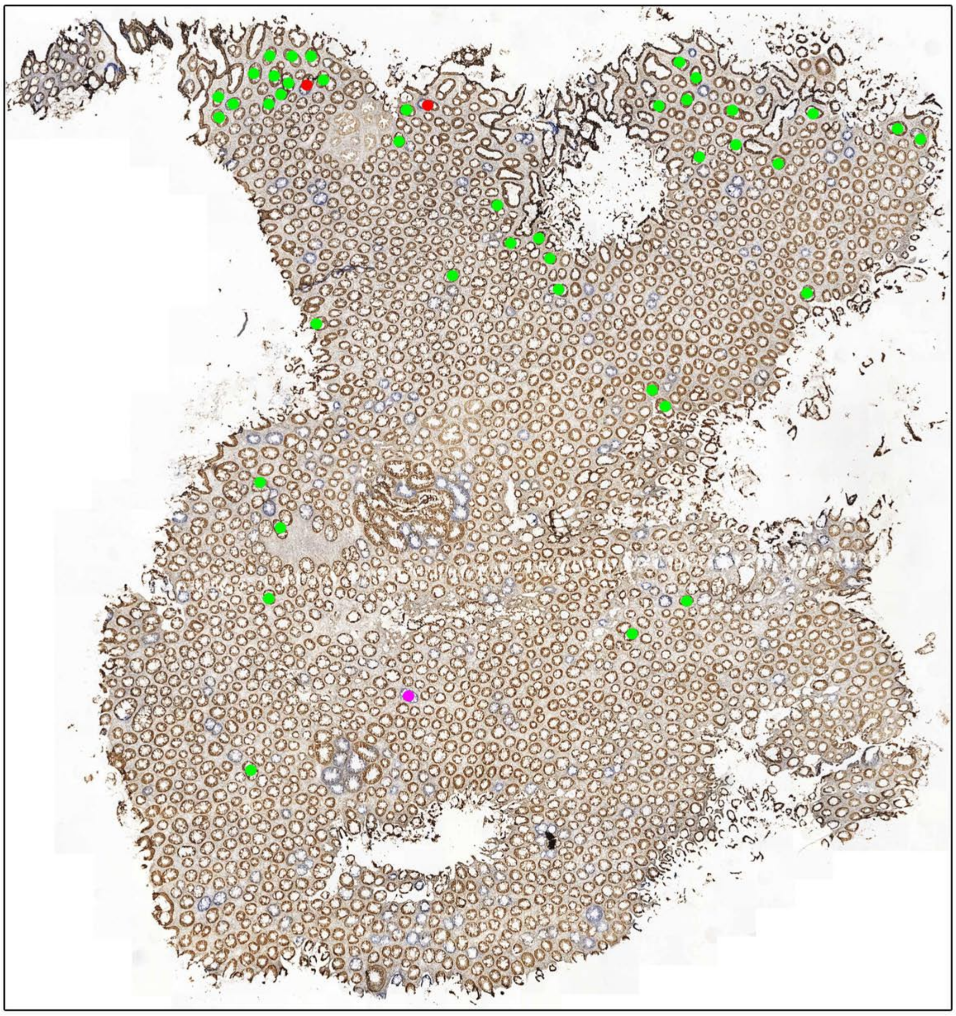

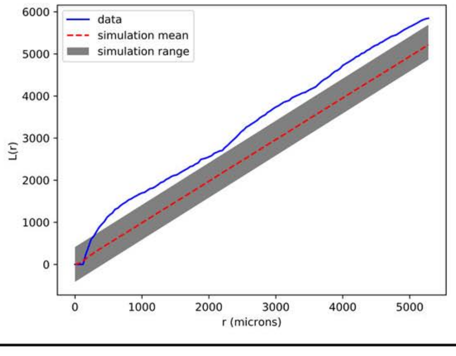

data
simulation mean
simulation range
6000
5000
4000
3000
2000
1000
0
L(r)
0
1000
2000
3000
4000
5000
r (microns)

Sample 5

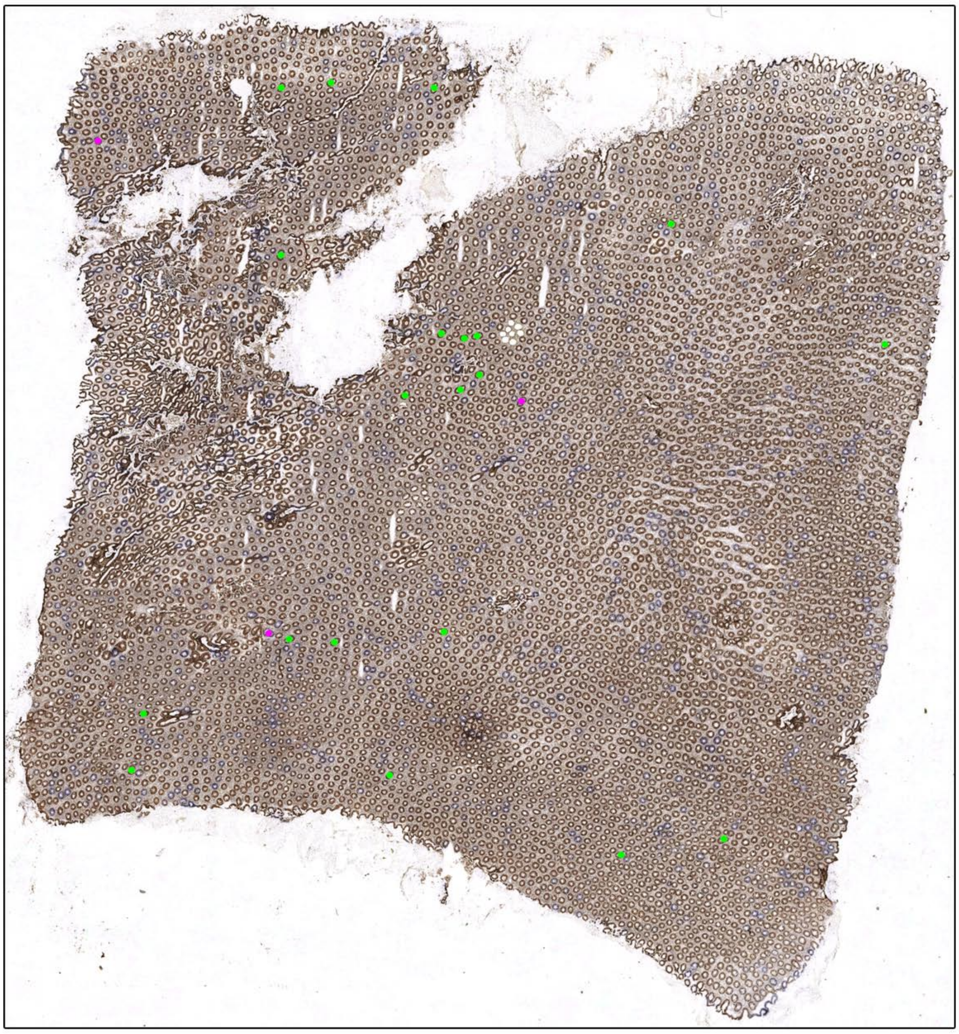

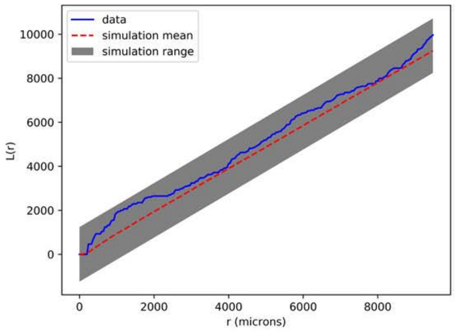

data
simulation mean
simulation range
10000
8000
6000
4000
2000
0
L(r)
0
2000
4000
6000
8000
r (microns)

Sample 6

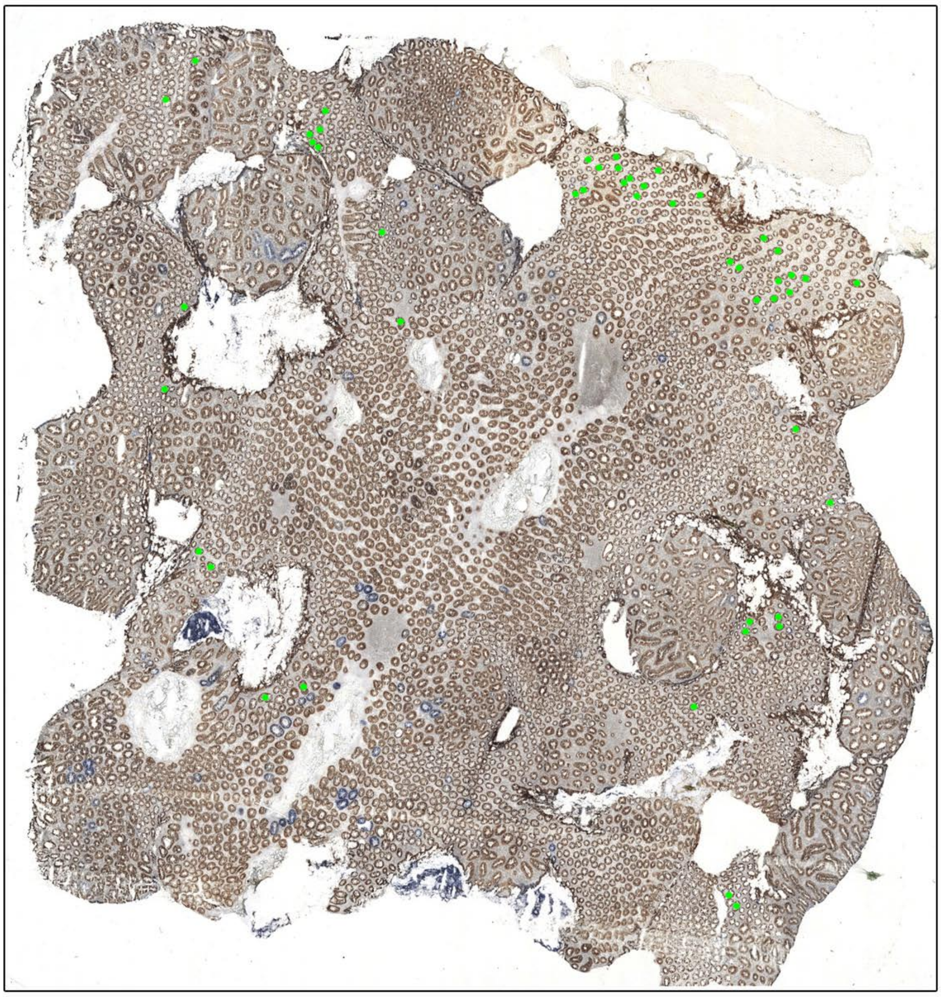

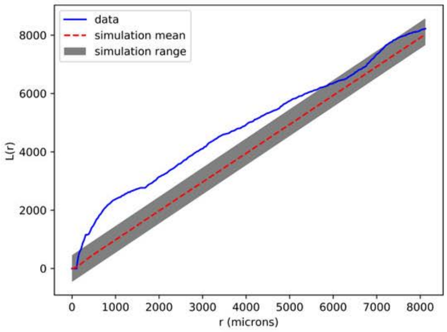
data
simulation mean
simulation range
8000
6000
4000
2000
0
L(r)
0
1000
2000
3000
4000
5000
6000
7000
8000
r (microns)

Sample 7a

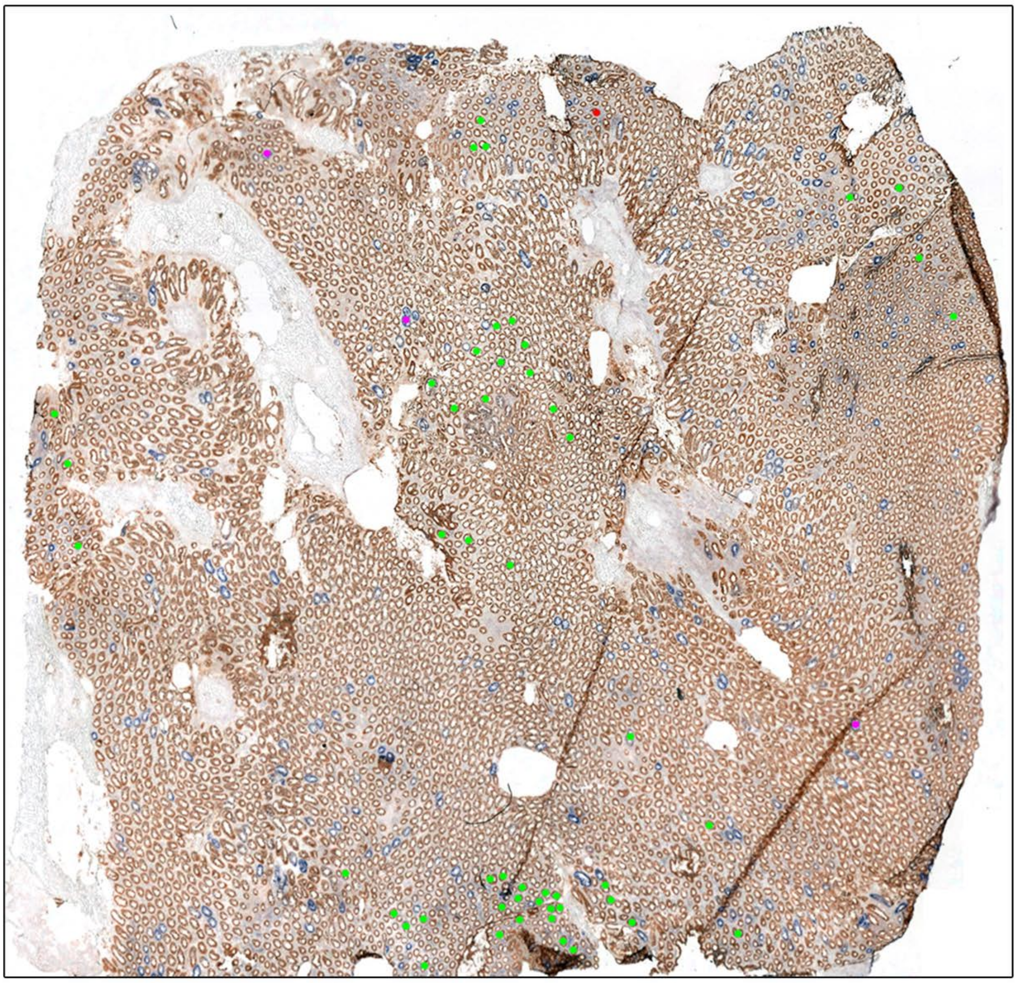

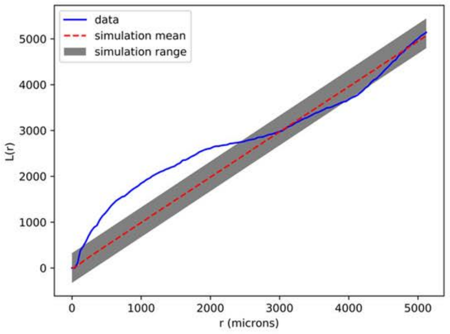
data
simulation mean
simulation range
L(r)
r (microns)
0
1000
2000
3000
4000
5000

Sample 7b

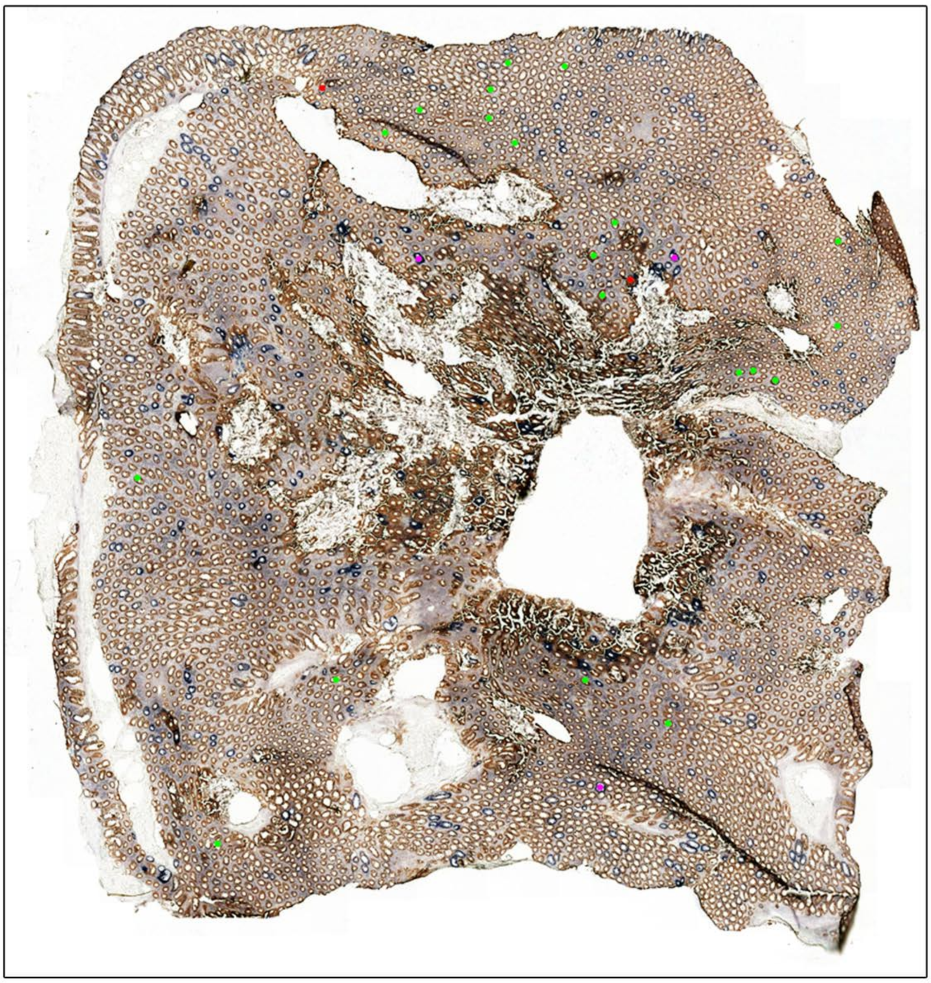

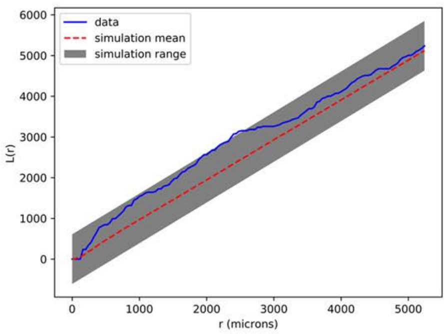

Sample 8

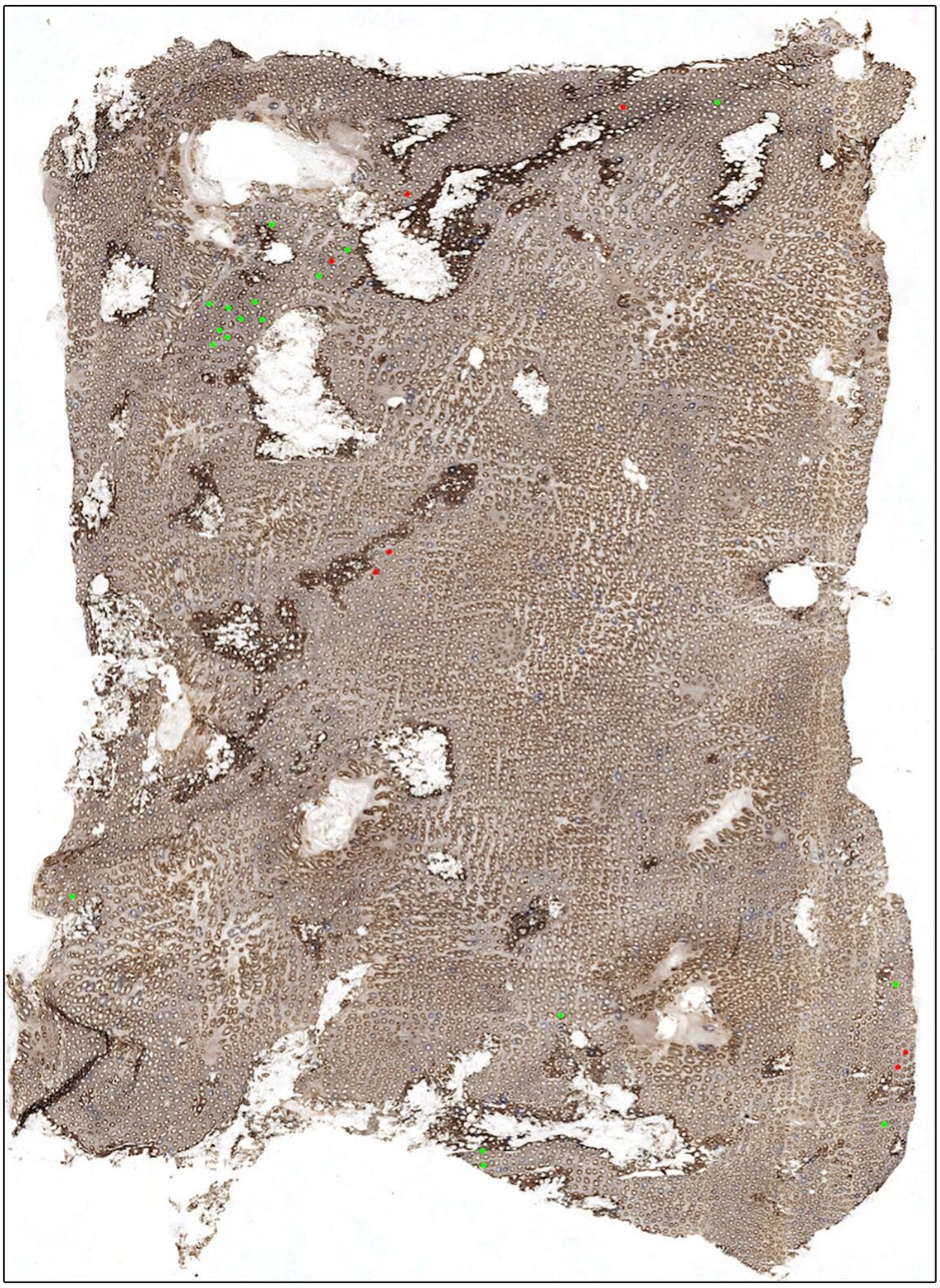

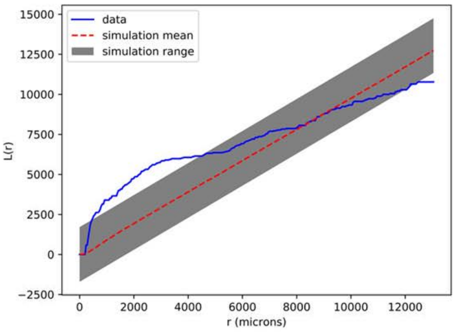

Sample 9

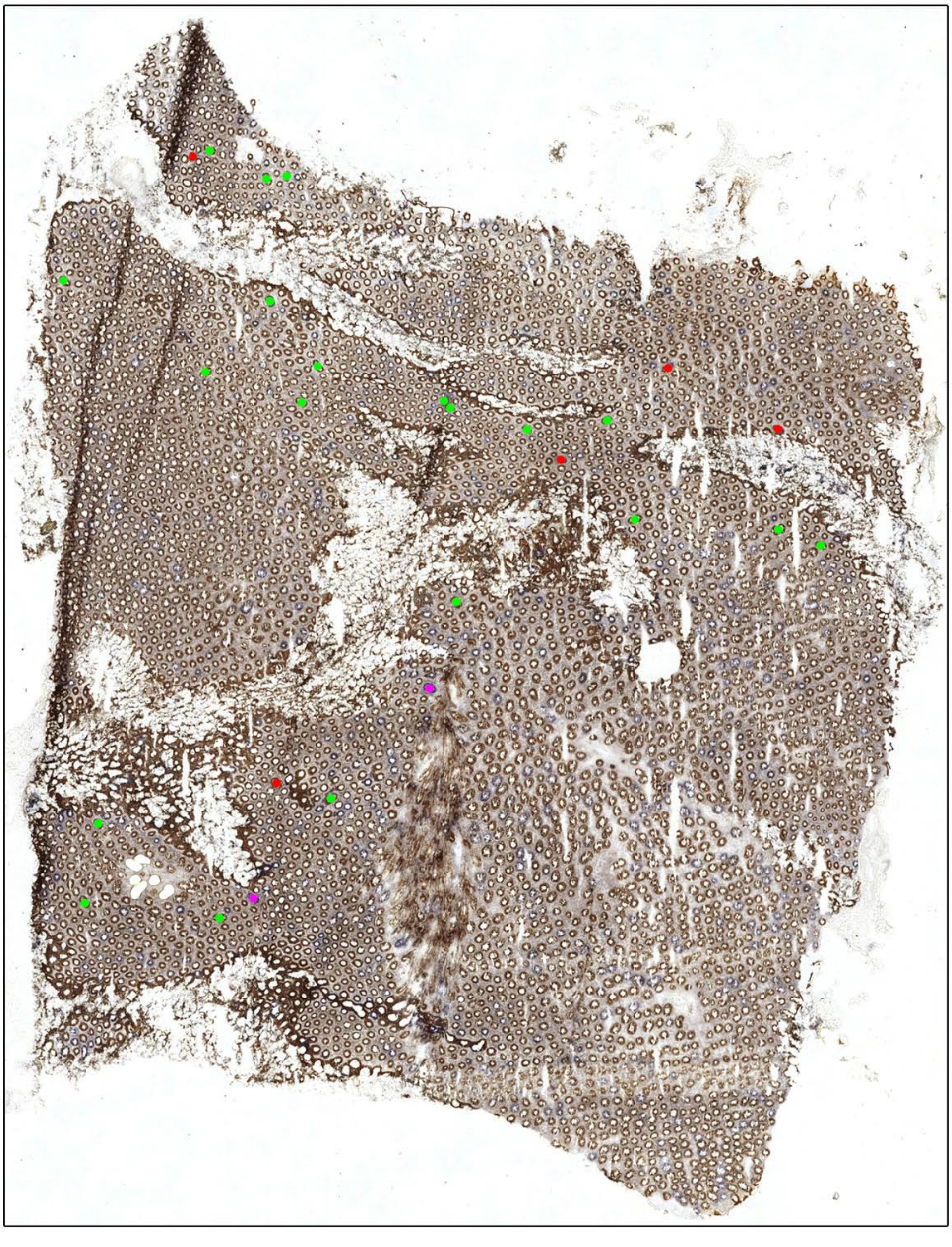

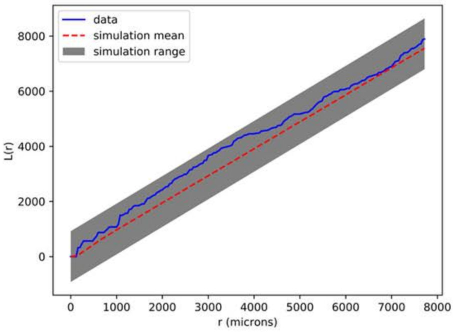
data
simulation mean
simulation range
L(r)
r (microns)
8000
6000
4000
2000
0
0
1000
2000
3000
4000
5000
6000
7000
8000

Sample 10

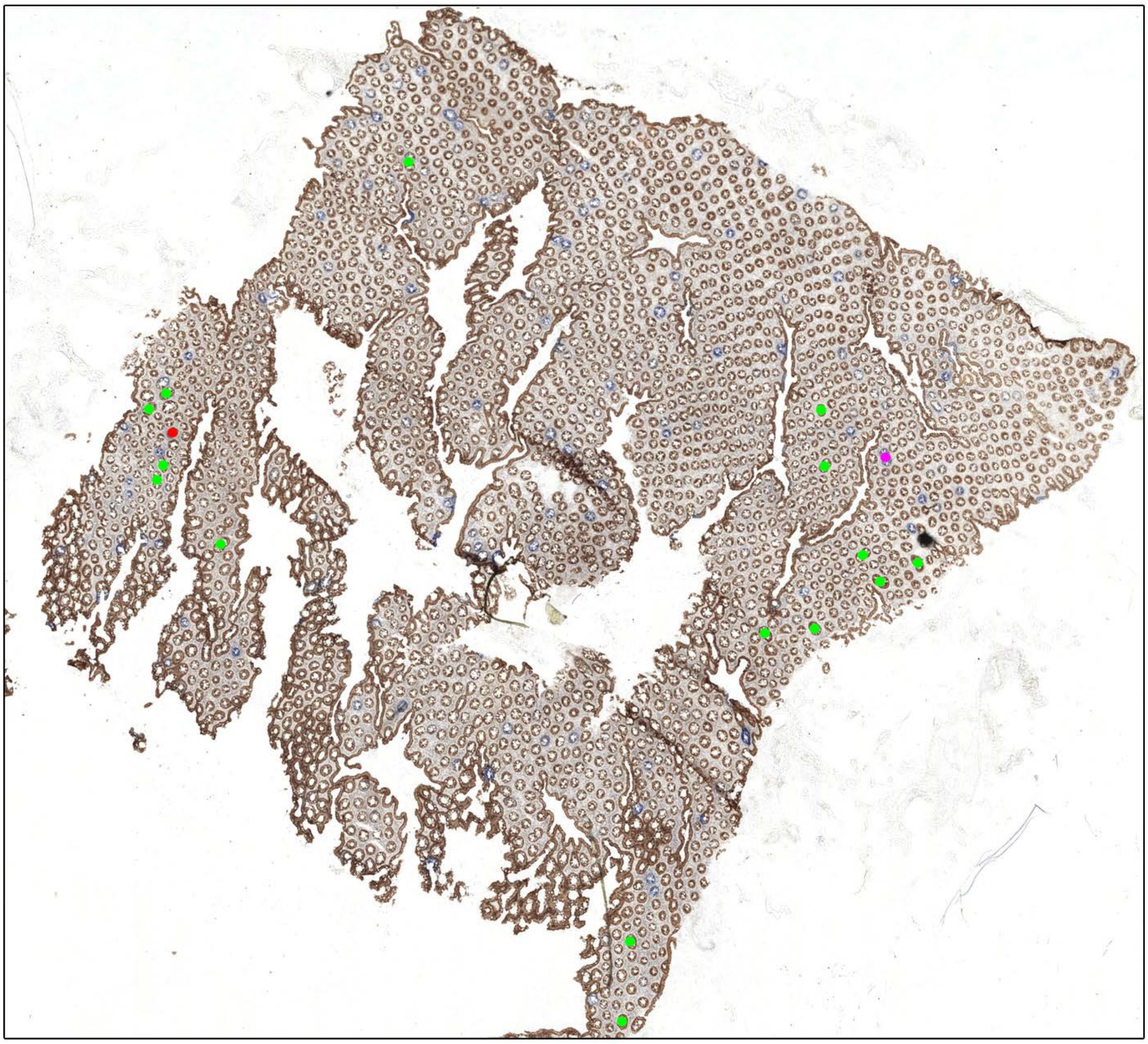

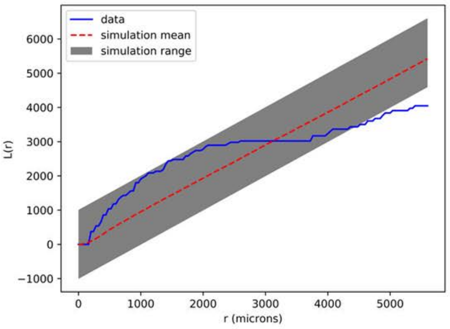

Supplementary Figure Legends

Supplementary Figure 1
Three additional representative examples of ‘Type III’ bifurcation events, showing the upper part of the crypts, in which the bifurcating pair clearly share a significant proportion of epithelium (right column), and the base of the crypts, in which they are clearly separate (left column). Scale bars represent 50 micrometres.

Supplementary Figure 2
A. 3D reconstruction of a mucosal subvolume showing a ‘Type III’ bifurcation event. The fusing crypts (CCO proficient in brown and CCO deficient in blue) are shown as spacefilling volumes and the surrounding crypts are shown as transparent wireframes. The accompanying Supplementary Video 1 shows the rotating subvolume.
B. Three individual sections reveal the individual legs of the bifurcating crypts, the ‘saddle point’ where both crypts merge, and finally the level at which both crypts have merged into a single orifice. Dashed lines indicate the fusing crypts in serial sections and numbers correspond to the surrounding crypts indicated in panel A. Scale bars represent 100 micrometres. The accompanying Supplementary Video 2 shows raw images of the serial sections before processing.

Supplementary Figure 3
Graphs showing the distribution of CCO-deficient patch sizes (black dots) and the maximum likelihood estimation (red line) for each patient.

Supplementary Figure 4
Representative images of each tissue section used for analysis of spatial correlation. Green dots represent Type I bifurcations, pink dots represent Type II events and red dots represent Type III events. Insets are graphs showing Ripley’s L function for the data from that sample (blue line), the mean of 999 simulations (red dotted line) and a global simulation envelope of significance at $\alpha=0.01$ (grey envelope).

Supplementary Video 1
Rotating 3D reconstruction of a mucosal subvolume showing a ‘Type III’ bifurcation event. The fusing crypts (CCO proficient in brown and CCO deficient in blue) are shown as spacefilling volumes and the surrounding crypts are shown as transparent wireframes.

Supplementary Video 2
Raw images of the serial sections used to produce the 3D reconstruction in Supplementary Video 1.

Supplementary Video 3
Raw images of serial sections showing a further example of a ‘Type III’ bifurcation event.

Supplementary Table 1 – Probability of perfect segregation by stem cell number

**Stem cell number (S)**	**Probability of perfect segregation at bifurcation**
5	1.49×10^{-21}
6	3.96×10^{-25}
7	3.98×10^{-28}
8	1.05×10^{-30}
9	6.91×10^{-33}
10	5.52×10^{-35}
